# Exploring the Health Effects of New Additive- and Allergen-Free Reformulated Cooked Meat Products: Consumer Survey, Clinical Trial, and Perceived Satiety

**DOI:** 10.3390/nu17101616

**Published:** 2025-05-08

**Authors:** Jhazmin Quizhpe, Pablo Ayuso, Fani Yepes, Domingo Miranzo, Antonio Avellaneda, Gema Nieto, Gaspar Ros

**Affiliations:** 1 Department of Food Technology, Nutrition and Food Science, Veterinary Faculty, University of Murcia, Regional Campus of International Excellence “Campus Mare Nostrum”, Espinardo, 30100 Murcia, Spain; jhazminedith.quizhper@um.es (J.Q.); pablo.ayuson@um.es (P.A.); gnieto@um.es (G.N.); 2 Cátedra de Seguridad y Sostenibilidad Alimentaria Grupo Fuertes, Universidad de Murcia, 30003 Murcia, Spain; fani.yepes@elpozo.com (F.Y.); domingo.miranzo@elpozo.com (D.M.); antonio.avellanedagoicuria@elpozo.com (A.A.)

**Keywords:** additives, cooked meat products, inflammation, oxidation, nitrates, gut microbiota

## Abstract

Background: Consumers are increasingly interested in healthier, less processed food products, driving the meat industry to improve the quality and health benefits of its offerings. Growing concerns about additives and allergens have encouraged the replacement of these ingredients with natural alternatives, presenting both challenges and opportunities. However, consumer rejection of additives and the actual health effects of their replacement remain poorly understood. In previous work, two new meat products—cooked turkey breast and cooked ham—were developed, where additives and allergens were replaced with natural extracts. These products demonstrated potential health benefits in vitro, including improvements in protein quality and microbiota composition. Methods: This study assessed consumer perceptions of additives through a survey and evaluated the two new meat products in a double-blind, randomized clinical trial conducted over a 5-week period. Biomarkers of interest were measured in blood, faeces, and urine samples at baseline and at the end of this study. Additionally, a separate study tested the satiating effect of these products using VAS score surveys. Results: The additive perception survey revealed that consumers associate additive-free products with being more natural and less harmful to health, with differences observed based on age, gender, and knowledge of additives. In the clinical trial, both the intervention and control groups showed significant decreases in serum levels of ox-LDL and GPx, with no differences between the groups. However, significant differences between the groups were found in inflammation markers TNF-α and IL-1β. Furthermore, the intervention group exhibited a significant reduction in nitrate excretion and a decrease in nitrification-related gut bacteria. Finally, the reformulated products demonstrated a satiating effect, reducing hunger. Conclusions: These findings suggest that the new additive- and allergen-free reformulated meat products may offer potential oxidative and anti-inflammatory benefits to consumers.

## 1. Introduction

Meat plays a fundamental role in a balanced human diet due to its high nutritional content. Meat products from beef, pork, or lamb are considered one of the main sources of high-quality protein, containing all the essential amino acids in adequate proportions [1]. Moreover, these products also contain essential omega-3 polyunsaturated fatty acids, minerals, such as iron and zinc, and vitamins, such as B_6_ and B_12_ [2,3]. Despite its nutritional benefits, concerns about the consumption of meat, especially red and processed meat, have increased significantly recently due to criticism from scientists and organizations [4]. Consequently, consumers are considering a reduction in the consumption of these products for a variety of reasons, such as the impact on the environment and their own health [5].

Additives are compounds added to food products to preserve their technological properties, avoid contamination by microorganisms, regulate acidity, and even act as stabilizers, thickeners, and emulsifiers [6]. Processed meat products include a wide variety of products prepared by some degree of alteration of the muscle structure through a variety of processes that can alter their properties [7]. For this reason, food additives are widely used in these products.

The health effects of additives in meat products remain controversial [8], partly because many of them have not been studied for long-term impacts. Not all additives have the same health implications. For instance, antioxidants, such as sodium citrate (E-331) and sodium erythorbate (E-316), as well as acidity regulators, like triphosphates (E-451), have not been reported to cause significant side effects or toxicity in healthy individuals [9,10]. However, some additives are more contentious. For example, carrageenan (E-407) is debated; while some studies suggest it may be beneficial for gut health [11], others associate it with intestinal inflammation and inflammatory bowel diseases in animal models [12,13]. Sodium nitrite (E-250) is another controversial additive. Although it may help regulate arterial hypertension through the nitric oxide pathway [14,15], its use in meat products is concerning due to its potential to form N-nitrosamines, which are known carcinogens [15].

The use and permissible amounts of food additives are strictly regulated worldwide, but regulations and authorities vary by country. In Europe, the European Food Safety Authority (EFSA) oversees the regulation of additives, permitting their use only in specific, non-hazardous amounts [16]. In the United States, the Food and Drug Administration (FDA) is responsible for federal regulation of these compounds [17]. Despite these stringent regulations, some consumers perceive additives as harmful and unsafe. Alongside concerns about salt content and saturated fat levels, the presence of additives is a major worry for many meat consumers [18]. While the reasons for the negative perception of additives are debated, studies suggest that consumer rejection is often due to their association with unnatural ingredients, potential toxicity, and a lack of understanding about their health effects [19,20,21].

The growing consumer demand for more natural products free from additives and allergens presents a significant challenge for the meat industry. To address this, the industry is focusing on reformulating processed meat products with natural ingredients. Recent advancements have enabled the replacement of additives, such as nitrates [22], phosphates [23], and synthetic antioxidants [24], with alternatives derived from citrus fruits and rosemary extracts. These changes aim to produce healthier options for consumers.

Additionally, there is a consumer preference for products that enhance satiety, as increased fullness can lead to appetite suppression and contribute to weight control [25,26]. Protein-enriched products are particularly valued for their satiating effects. Research indicates that dietary proteins influence gastrointestinal responses that reach the brain through both direct humoral and indirect neural pathways [27].

In this context, previous research [28] developed two new meat products that exclude additives and allergens, incorporating instead a blend of natural extracts, such as vitamin C, chlorogenic acids, hydroxytyrosol, catechins, epicatechins, vinegar, and inulin fibre. In vitro results from the study suggest potential health benefits related to protein quality and gut microbiota.

Given the increasing concern regarding the use of additives in meat products, it is crucial to investigate both the underlying reasons for consumer aversion and the potential health benefits of consuming processed meat products free from additives and allergens. The hypothesis of this study posits that additive-free meat products may be more appealing to consumers and may provide associated health benefits.

To test this hypothesis, two additive- and allergen-free meat products (cooked ham and cooked turkey breast), which had previously demonstrated potential health benefits in vitro [28], were developed. The primary aim of this research was to assess consumer attitudes towards additives, with a particular focus on meat products, through a perception survey. Following this, this study aimed to evaluate the effects of the regular consumption of these new additive- and allergen-free meat products through a short randomized clinical trial. This trial assessed the impact on a range of biomarkers, including routine biochemical markers, anti-inflammatory markers, and oxidative stress markers in blood, as well as microbiota composition and metabolic activity in faeces. Additionally, nitrogen metabolism in urine was analysed. To further complement these findings, an appetite and satiety assessment was conducted, involving different participants for each aspect of the trial.

## 2. Materials and Methods

### 2.1. Food Additives Questionnaire

A questionnaire was developed (Supplementary Material File S1 Additives Perception Survey) to collect data from a large population on the perception of food additives. The survey consisted of 27 questions concerning the purchase of processed foods, as well as the basic knowledge and perception of the safety of food additives, particularly in meat products. For this purpose, the survey was divided into four different blocks: demographic data of the participants, general questions about additives, influence of additives when purchasing a food product, and perception aspects of additives in meat products. The responses were analysed with both multiple-choice and 10-point Likert scale questions. The survey was designed in an online format using an online survey platform of the University of Murcia and was available for completion during the first trimester of the year 2023.

A total of 330 participants completed the questionnaire and were categorized based on demographic factors, such as gender, age, allergen status, and education level. The demographic breakdown and corresponding participant numbers are provided in Table 1.

### 2.2. Reformulated and Commercial Meat Products

Four different cooked meat products were used, which were manufactured and supplied by a local Spanish company (ElPozo Alimentación, S.A., Alhama de Murcia, Murcia, Spain). These products were a standard cooked turkey breast (ST) and a reformulated cooked turkey breast (RT), as well as a standard cooked ham (SH) and a reformulated cooked ham (RH). The additives, including E-451 (triphosphates), E-407 (carrageenan), E-420 (sorbitol), E-316 (sodium erythorbate), E-331 (sodium citrate), and E-250 (sodium nitrite), and the allergens, including milk and soya, from the standard products were replaced by a 0.3% mix of natural herbal extracts, rich in vitamin C, chlorogenic acids, hydroxytyrosol, catechins, and epicatechins, by 0.3% vinegar, and by 3% inulin fibre to obtain the new reformulated products. The cooked meat products used in this study, which were free of additives and allergens, were developed and analysed in a previous in vitro study [28]. The results showed that these products obtained a higher digestibility and protein quality without altering the composition of the intestinal microbiota and increasing the production of short-chain fatty acids. The nutritional composition of the products is detailed in our previous work [28].

### 2.3. Randomized Clinical Trial (RCT)

#### 2.3.1. RCT Study Design and Approval

To evaluate the potential effects of consuming the reformulated, additive- and allergen-free cooked meat products on metabolism and health—specifically, antioxidant and inflammatory markers, gut microbiota composition, and metabolic indicators of intestinal well-being—a five-week, double-blind, randomized, controlled, unicentric trial was conducted with two parallel groups (control vs. reformulated products). This study was approved by the Bioethics Committee of the University of Murcia (UMU) under code 550/2023, in accordance with the Declaration of Helsinki, and was registered at ClinicalTrials.gov under the identifier NCT06392893 in May 2024. To ensure transparency and completeness in reporting, we followed the CONSORT standards (“Consolidated Standards of Reporting Trials”) and included the CONSORT checklist as Supplementary Material File S2 CONSORT 2010 Checklist. The CONSORT checklist Item Nos. are indicated in the heading sections and subsections.

This research was conducted at the Department of Nutrition and Bromatology, Faculty of Veterinary, University of Murcia. All clinical analyses and participant management were carried out by the Medical Service of the University of Murcia. The participants were blinded to group assignment, and the allocation sequence was concealed from the investigators (double-blind design). The cooked meat products used in the trial included control and reformulated versions of turkey breast and cooked ham, delivered in 100 g blister packs.

#### 2.3.2. Participant Recruitment and Allocation

**Figure 1:**
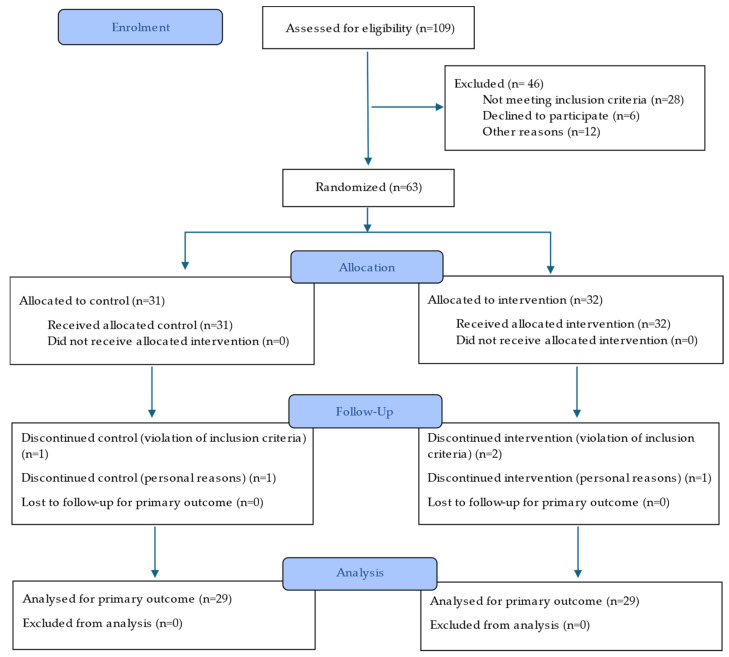
CONSORT flow diagram of the intervention trial.

Recruitment began three weeks before the intervention, during which eligible candidates received detailed information about the study protocol. Out of 63 individuals initially recruited, 58 completed the trial (Figure 1). The participants were randomly allocated to one of two groups (control or reformulated) by an external researcher using a balanced distribution according to age, sex, and BMI. The subjects attended two visits: one at baseline and one at the end of the intervention. During both visits, blood, faecal, and urine samples were collected, and anthropometric measurements were recorded.

The participants received two deliveries of 15 blister packs each (a total of 30), with 1 blister (100 g) consumed daily for six days a week. The turkey breast and cooked ham were alternated daily. Dietary intake was recorded by each participant throughout the trial to monitor potential dietary interferences.

#### 2.3.3. Participants and Random Allocation

The participants were randomly assigned to the intervention and control groups using a computer-generated simple randomization sequence in a 1:1 ratio. The allocation sequence was generated by an independent researcher not involved in participant enrolment, and group assignments were concealed using sealed opaque envelopes to maintain allocation concealment throughout the process. Fifty-eight subjects were included in the final analysis, divided into two balanced groups of twenty-nine participants each (Table 2). The inclusion criteria were age 18–65 years and BMI between 18.5 and 30 kg/m^2^.

The exclusion criteria included recent use of antibiotics, medications for hypertension or hyperlipidaemia, dietary supplements (including prebiotics, probiotics, and omega-3/6), the presence of eating disorders, chronic or infectious diseases, pregnancy, smoking, adherence to a vegetarian diet, recent surgery or blood donation, and participation in another clinical trial within the preceding three months. Although none of the enrolled participants met any of the exclusion criteria, five individuals withdrew from this study for personal reasons.

#### 2.3.4. Demographic and Anthropometric Assessments

Anthropometric measurements were performed according to ISAK protocols [29]. These included height, weight, BMI, body fat percentage, and circumferences (waist, hip, abdomen), measured with validated tools (e.g., OMRON BF306 and Seca anthropometric tape). The waist-to-hip ratio (WHR) was calculated accordingly (cm waist–cm hip).

#### 2.3.5. Sample Collection and Analysis

Twelve-hour fasting blood samples were drawn from the antecubital vein in gel and clot activator tubes (Vacutest Kima Srl, Arzergrande, Italy) and then processed (centrifuged at 3500 rpm for 5 min at 4 °C to separate the serum from the cell fraction). After collection, the serum portions were stored at −80 °C until analysis.

##### Biochemical Parameters

Glucose, glutamate oxaloacetate transaminase (GOT), glutamate pyruvate transaminase (GPT), high-sensitivity C-reactive protein (hs-CRP), and lipid markers (triglycerides (TGs), low-density lipoprotein cholesterol (LDL-C), high-density lipoprotein cholesterol HDL-C, and total cholesterol (TC) were measured using a clinical chemistry analyser (BA400 Biosystems, Biosystems S.A., Barcelona, Spain).

##### Inflammatory Markers and Oxidative Stress Markers

Interleukin (IL)-1β, IL-6, IL-10, and tumour necrosis factor-alpha (TNF-α) were analysed using commercially available kits (Milliplex Human High Sensitivity T Cell; Life Science, Darmstadt, Germany) according to the manufacturer’s instructions. Oxidative biomarkers were measured using validated clinical chemistry analysers and ELISA kits. Catalase activity was assessed by an automatic method previously described by Slaughter et al. [30]. FRAP measurement was based on the method described by Benzie and Strain [31], with some modifications [32]. Glutathione peroxidase (GPx) was determined using a commercially available method (Ransel, Randox Laboratories Limited, Crumlin, UK). FRAP and GPx were measured in an automated chemistry analyser (Olympus AU400, Olympus Diagnostica Europe GmbH, Ennis, Ireland). The TBARS assay was determined following the method by Buege and Aust [33]. Oxidized Low-Density Lipoprotein (ox-LDL) was determined using a commercially available ELISA Kit (Human ox-LDL (Oxidized Low-Density Lipoprotein) ELISA Kit, Houston, TX, USA) [34]. TBARS and ox-LDL were measured using a microplate reader (Powerwave XS, Biotek instruments, Winooski, VT, USA).

##### Sample Preparation for FRAP and Short-Chain Fatty Acid Analysis

Faecal samples were thawed at 4 °C for 24 h and then diluted 1:10 with 0.1 M phosphate buffer (2.33 g Na_2_HPO_4_ and 14.03 g NaH_2_PO_4_ in 2 L of water, pH 7.4) and sterilized. The mixture was centrifuged for 20 min at 5000 rpm to collect the supernatant, which was centrifuged again for 10 min. The final supernatant was separated into two fractions for SCFA and FRAP assay analysis. The SCFA sample was stored at −80 °C until use, while the FRAP assay was performed on the same day as the sample preparation.

The antioxidant capacity of the faecal samples was measured using the FRAP assay, following the method described by Benzie and Strain [31]. For this, the supernatant was diluted 1:10 with water.

SCFA production was assessed following the method outlined by Panzella et al. [35], with some modifications. The supernatant was filtered through 0.20 µm PTFE filters and analysed by reversed-phase HPLC with a diode array detector (1260 Infinity II LC System, Agilent, Santa Clara, CA, USA). The results were obtained in ppm, converted to mmol/kg of faeces, and expressed as the increase in SCFAs over 24 h.

##### DNA Extraction and Metagenomic Sequencing

Bacterial genomic DNA was extracted from stool samples using the NZY Soil gDNA Isolation Kit (Nzytech, Lisboa, Portugal). Sequencing of the V3 and V4 regions of the 16S rRNA gene was performed on the MiSeq platform (Illumina, Essex, UK) with 2 × 300 bp reads. The oligonucleotide primers targeting the 16S rRNA V3 and V4 regions were 5′-TCGTCGGCAGCGTCAGATGTGTATAAGAGACAGCCTACGGGNGGCWGCAG-3′ and 5′-GTCTCGTGGGCTCGGAGATGTGTATAAGAGACAGGACTACHVGGGTATCTAATCC-3′, respectively [36].

The 16S-V4 sequencing libraries were initially assessed for quality using FastQC v0.12.1, followed by processing with the DADA2 R package (v.1.8.0.) [37]. The reads were quality-trimmed using the “filterAndTrim” function with “maxEE (2.5)”, and those shorter than 165 bp were discarded. Forward and reverse reads were merged to generate a sequence table, and Amplicon Sequence Variants (ASVs) were subjected to de novo chimera detection in DADA2, with any artifacts removed.

##### Bioinformatic Analysis

For bacterial taxonomic assignment, ASVs were queried against the Silva database v.132 [The SILVA ribosomal RNA gene database project: improved data processing and web-based tools] using IDTAXA [38] implemented in the R package DECIPHER. Sequences identified as non-bacterial were discarded.

The abundance matrix, taxonomy assignments, and sample metadata were merged and imported into the phyloseq package (v3.12) [39]. Alpha diversity was calculated in R using phyloseq, with several indices generated, including ASV numbers, Chao1, ACE, Shannon, Simpson, InvSimpson, and Fisher, and visualized using the “plot_richness” function. Beta diversity was calculated based on weighted and unweighted UniFrac distances [40]. To assess significant differences in community composition across different treatments and time, permutational multivariate analysis of variance (PERMANOVA) was performed using distance matrices with the Adonis function in R, and the results were visualized through Principal Coordinates Analysis (PCoA).

##### Nitrate and Nitrite Analysis

The participants provided first-morning urine samples, which were refrigerated and delivered to the laboratory within 24–48 h. The samples were then stored at −20 °C until further use. The urine samples were analysed for nitrate and nitrite content. The samples were first diluted 1:10; then, 1 mL of Carrez I (K4[Fe(CN)6]·3H_2_O) and 1 mL of Carrez II (ZnSO_4_·7H_2_O) were added, and the mixture was stirred for 30 min at room temperature. After stirring, the mixture was filtered through a 150 mm diameter filter paper. The filtered samples were then analysed using an autoanalyzer (Y15, Biosystems, Barcelona, Spain).

### 2.4. Palatability and Satiety Evaluation

#### 2.4.1. Participants and Study Design

This study employed a randomized, crossover design, in which each participant consumed three different breakfast interventions (basal, control, and reformulated), with a one-week washout period between each session to minimize carryover effects. All information regarding this evaluation can be found in Supplementary Material File S3 Satiety Evaluation.

Eligible participants were healthy adults aged 18 to 60 years (mean ± SD: 30.95 ± 12.77 years) with a body mass index (BMI) between 20 and 30 kg/m^2^ (mean ± SD: 25.19 ± 4.04 kg/m^2^). The exclusion criteria included being a professional athlete, current smoking, regular use of prescription medications (except for oral contraceptives), and pregnancy. Female participants were excluded during their menstrual period to minimize potential hormonal influences on satiety-related variables.

All the participants were fully informed of this study’s objectives, procedures, duration, and potential risks and benefits. Written informed consent was obtained from all individuals prior to enrolment.

The basal breakfast (370 kcal) consisted of 250 mL of pineapple juice, 70 g of toasted bread, 100 g of grated tomato, and 10 g of extra virgin olive oil. Both the control (410 kcal) and reformulated breakfasts (416 kcal) were based on the same basal meal, with the addition of 25 g of cooked turkey breast and 25 g of cooked ham. The control breakfast included conventional meat products, while the reformulated version featured meat products developed with natural ingredients [27].

The participants were instructed to consume their usual evening meal before each test day and to fast for at least 12 h overnight. Additionally, they were advised to abstain from consuming any other food or drink (except water) during the 3 h study period, to avoid alcohol intake, and to refrain from engaging in vigorous physical activity for 24 h prior to each intervention. The detailed nutritional compositions of the breakfast meals are presented in Table 3.

#### 2.4.2. Appetite and Satiety Evaluation

Satiety was evaluated using standardized visual analogue scales (VASs) [41], a validated tool for subjective appetite assessment. Each VAS survey consisted of 13 items addressing sensations such as hunger, fullness, desire to eat, expected food consumption, nausea, and bloating. For each item, the participants marked their response on a 10 cm horizontal line anchored with opposing descriptors (e.g., “not at all” on the left and “very/extremely” on the right), reflecting the intensity of their sensation.

The VAS surveys were administered at six time points: immediately before breakfast (baseline), immediately after consumption (time 0), and at 45, 90, 135, and 180 min postprandially. The participants were instructed to refrain from eating or drinking (other than water) until all postprandial assessments were completed.

The quantitative analysis of satiety included the calculation of the area under the curve (AUC) for each variable across the 3 h period, the initial response (difference between post-breakfast and pre-breakfast scores), and the incremental VAS score at 180 min (VAS_180_) to assess the persistence of satiety effects over time.

### 2.5. Statistical Analysis

The data are presented as mean ± standard deviation (SD). To assess within-group changes over time (baseline vs. final), Student’s *t*-test was applied. To evaluate differences between groups in the progression of variables, a repeated measures analysis of variance (ANOVA) was conducted, with time (baseline and final) as the within-subject factor and treatment group (control vs. intervention) as the between-subject factor. Tukey’s post hoc test was used for pairwise comparisons when significant effects were detected.

Perceptions regarding food additives were analysed using cross-tabulations and chi-square (χ^2^) tests, based on demographic variables. The area under the curve (AUC) for satiety-related outcomes was calculated using the trapezoidal rule [42].

All data processing and statistical analyses were performed using SPSS version 28.0 (IBM Corp., Armonk, NY, USA). Statistical significance was set at *p* ≤ 0.05.

## 3. Results

### 3.1. Food Additives Survey

Before initiating the clinical trial, a general survey on additives was conducted. The primary aim of this questionnaire was to gather perception data on food additives from a homogeneous population through straightforward yes/no or multiple-choice questions. The general perceptions of additives are summarized in Table 4.

In response to the question, “What preference would you have for two products of similar taste and price that differ only in the presence of additives?”, 71.3% of the respondents preferred to buy a product without additives, while only 26.1% were indifferent to the presence of additives. Additionally, 78.2% of the respondents viewed a product as more “natural” if it did not contain additives. These findings align with those of Roman et al. [19], who reported that consumers consider the presence of artificial ingredients, preservatives, additives, chemicals, hormones, or pesticides as factors that make a product appear less natural.

Conversely, one reason respondents might prefer additive-free products is the perception that these products are less toxic. Up to 45.8% of the respondents chose this reason over “improves intestinal health” (15.8%) or “they have no benefits” (13.6%). Additionally, the primary perceived benefit of additives is that “they help to better preserve the food” (61.5%). This suggests that while the respondents acknowledge the functional role of additives in preservation, they remain concerned about their potential toxicity. This scepticism towards additives has been documented in other studies; for example, a study in Korea found that 76.7% of respondents considered government-approved additives to be unsafe [43], primarily due to insufficient data on their safety.

To test perceptions of food additives against other demographic parameters, the question “What preference would you have for two products of similar taste and price that differ only in the presence of additives?” was stratified by parameters such as gender, age, and knowledge of additives. The results are shown in Table 5.

### 3.2. Clinical Trial

#### 3.2.1. Body Composition Measurements

Table 6 provides a detailed summary of the anthropometric measurements for all the participants at both the beginning and end of the trial. No significant differences were observed in any anthropometric parameters between the control and intervention groups after 5 weeks of consuming the respective cooked meat products. Specifically, the regular consumption of cooked turkey breast and cooked ham did not result in changes in weight, BMI, body fat percentage, abdominal circumference, or ICC. This suggests that the daily intake of these products does not lead to increases in weight or body fat percentage. Furthermore, there were no significant differences between the control and intervention groups for any of these parameters post-intervention. This indicates that replacing additives and allergens with natural extracts does not impact body composition, at least at the administered dose.

#### 3.2.2. Blood and Urine Biomarkers

Blood serum and urine samples were analysed at both baseline and the end of the trial for both the control and intervention groups. The results for the glycaemic, lipid, oxidative, inflammation, and additive exposure parameters are presented in Table 7 as means and standard deviations (means ± SDs).

After 5 weeks of nutritional intervention with the new additive- and allergen-free products, no significant changes were observed in the lipid and glycaemic markers. Although a trend towards decreased blood triglycerides was noted with the reformulated product, the results were not statistically significant.

Significant differences, however, were found in certain oxidation markers in both groups. For serum ox-LDL, the intervention group showed a significant reduction from baseline (233.6 ± 52.4) to the end of the trial (192.4 ± 29.9) (*p* < 0.05). Similar significant changes were observed in the control group (238.6 ± 52.1 to 193.0 ± 40.2), with no significant differences between the groups, suggesting that both products similarly impact this marker.

For serum glutathione peroxidase (GPx), the intervention group exhibited a significant decrease from baseline (436.5 ± 172.0) to the final values (311.3 ± 59.6). Significant reductions were also noted in the control group (491.6 ± 382.9 to 340.8 ± 76.9), but no significant differences were detected between the groups. These changes in oxidation markers may be attributed to the high content of phenolic compounds and antioxidant capacity present in both the control and reformulated products, as detailed in our previous study [28].

#### 3.2.3. Gut Microbiota

**Figure 2:**
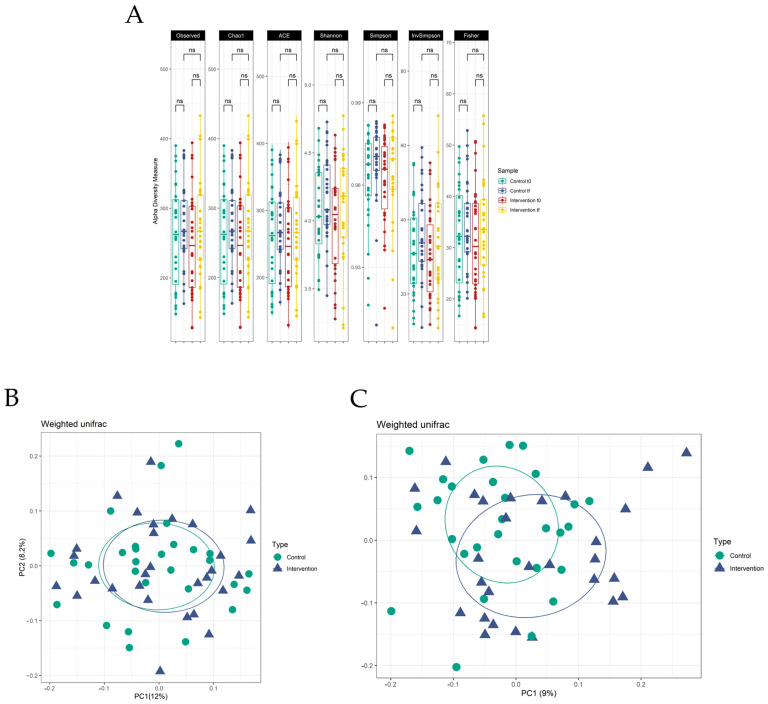
Effect of the reformulation on the alpha and beta diversity of microbial communities. Changes in the gut microbiota α-diversity of both groups (**A**). Different indices were used for measurement: Observed, Chao1, ACE, Shannon, Simpson, Inverse Simpson, and Fisher. The error bars represent the SD of each group (*n* = 29). Significant differences are indicated (ns: non-significant). Changes in the gut microbiota β-diversity for the initial time (**B**) and final time (**C**) using UniFrac Weighted distances. The axes represent the two dimensions explaining the greatest proportion of variance in the communities for each analysis. Each shape is a participant in each group.

The microbial community structure of the control and intervention groups was analysed using the faecal samples of the participants. To achieve this, the alpha and beta diversity, as well as the relative abundance of the bacterial communities, were assessed. The results pertaining to the microbial community composition are presented in detail in Figure 2.

##### Alpha and Beta Diversity

Regarding intracommunity bacterial diversity (alpha diversity) (Figure 2A), no significant differences were observed between the initial and final times for both the control and intervention groups. Furthermore, no significant differences were observed between the final times for the two groups. These findings were repeated for all the indices utilized in this study. These findings indicate that the regular consumption of cooked meat products over a five-week period does not result in alterations to the intracommunity diversity of the gut microbiota.

Additionally, the intercommunity diversity of gut bacteria (beta diversity) was examined using weighted UniFrac distances in the PCoA ordination analysis. The initial differences between the two groups of participants were compared (Figure 2B), and it was observed that the populations were very similar to each other, showing no clear difference in the PCoA plot. In contrast, for the final time samples (Figure 2C), the two groups were observed to be more separated.

To elucidate the alterations in intercommunity diversity, an analysis of the microbial composition at varying levels within both groups was conducted.

##### Relative Abundance

**Figure 3:**
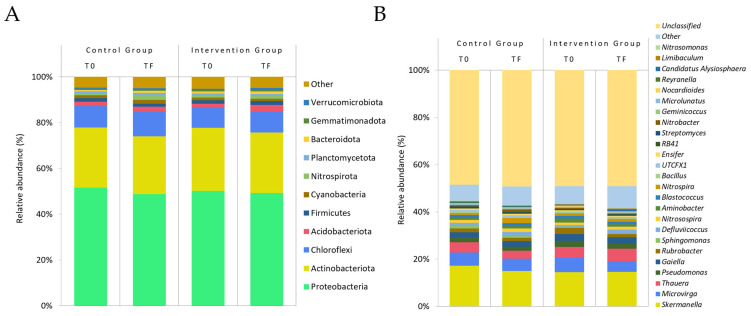
Bar-plot of gut microbial community structure at the phylo (**A**) and genus (**B**) level in percentage of relative abundance in both the control and intervention groups. T0: initial time; TF: final time.

The bacterial composition at the phylo and genus levels is illustrated in Figure 2B and Figure 3A, respectively. Table 8 provides a detailed account of the significant differences in the relative abundance observed at both the phylo and genus levels between the initial and final time points for each group, as well as between the groups.

At the phylo level, there was an increase in Acidobacteriota in both the control group (1.940 ± 1.607 to 2.201 ± 1.612) and the intervention group (1.621 ± 1.794 to 2.948 ± 2.740). However, this increase was only significant in the latter group. Another noteworthy alteration was observed in Nitrospirota, where significant differences between the groups were identified for this phylo, exhibiting a marked increase in the control group (0.877 ± 0.859 to 2.197 ± 2.922) and notable stability in the intervention group (1.135 ± 1.213 to 1.313 ± 1.478).

#### 3.2.4. Faecal Markers

Additionally, the antioxidant capacity (FRAP) and the production of short-chain fatty acids (acetic, propionic, and butyric acid) were evaluated in the faecal samples obtained from the participants. The results are presented in detail in Table 9.

Regarding the faecal antioxidant capacity, no significant differences were observed within or between the groups. Furthermore, no notable alterations were discerned in the generation of short-chain fatty acids after the ingestion of the products. For each of these compounds, whether considered individually or in the context of the overall sum, a downward trend was observed, with a greater decline evident in the control group than in the intervention group.

### 3.3. Satiety Assay

**Figure 4:**
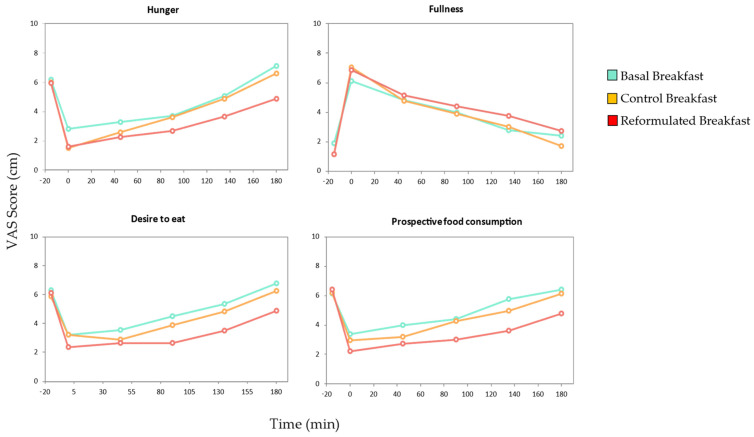
Subjective appetite scores of the three different breakfasts. Data are presented as mean values (n = 21).

The VAS scale appetite and satiety survey enabled the researchers to obtain data regarding the volunteers’ hunger, fullness, desire to eat, and prospective consumption levels during this study. Figure 4 illustrates the data pertaining to this satiety assay. By employing the data obtained from the VAS surveys, it was possible to estimate the subjects’ appetite and satiety levels by calculating the area under the curve (AUC), the initial VAS score, and the increment in the VAS score at 180 min (AUC_180_) (Table 10).

## 4. Discussion

The chi-square analysis revealed no significant differences in additive preference by sex. However, age was found to be a significant factor influencing the perception of additives. The results indicated that the respondents from the Baby Boomer generation (1946–1967) and Generation X (1968–1978) placed greater importance on the absence of additives in their food compared to Generation Z (1998–2012) and Millennials (1979–1997). A study on the Australian population [44] similarly found that individuals over 45 years of age were significantly more concerned about the toxicity of additives compared to those aged 18–35 years. Additionally, the rejection of additives was notably higher among those aged 56–65 and over 65, compared to those aged 46–55, suggesting an increased concern about food safety risks with age.

Another noteworthy finding from the survey is the significant association between knowledge about additives and indifference to their presence in products. Among the participants who claimed to have knowledge about food additives, 34.4% were indifferent to their content, while only 18.5% of those without such knowledge were indifferent. These results suggest that consumers unfamiliar with food additives are more concerned about their use in processed foods.

Given the negative reputation of additives and their perception as toxic and harmful, it is crucial to explore their actual impact on health, particularly within the context of meat products.

Both the intervention and control participants maintained waist-to-hip ratios (WHRs) below the established cardiovascular risk cut-off [45], and no significant intergroup differences were detected. These results indicate that the regular consumption of either the reformulated or control cooked meat products did not alter central adiposity, challenging common associations between processed meat intake and increased obesity risk [46,47].

The observed reduction in oxidized low-density lipoprotein (ox-LDL) is particularly noteworthy, as LDL oxidation within the vascular endothelium represents a key initiating event in atherogenesis and markedly increases cardiovascular risk [48]. The augmented antioxidant content of the reformulated products—comprising vitamin C, chlorogenic acids, hydroxytyrosol, catechins, and epicatechins—likely contributed to this decline, mirroring the protective effects traditionally attributed to synthetic antioxidants (e.g., E-316, E-331) [49].

Conversely, glutathione peroxidase (GPx) activity, an intracellular enzyme that catalyses the reduction of hydrogen peroxide to water and thereby mitigates oxidative damage [50], was lower following the consumption of the reformulated meats. This decrease may reflect partial substitution of endogenous enzyme function by the exogenous antioxidants supplied in the diet.

With respect to inflammation, serum interleukin-1β (IL-1β) remained essentially stable in the intervention group (3.96 ± 2.74 to 4.01 ± 2.13 pg/mL), whereas it rose significantly in the control group (3.27 ± 1.64 to 4.68 ± 3.68 pg/mL; *p* < 0.05) [51,52]. A similar pattern was observed for tumour necrosis factor-α (TNF-α), which decreased in the intervention arm (16.56 ± 3.97 to 14.08 ± 2.66 pg/mL) but not in the control arm (16.36 ± 5.44 to 16.94 ± 4.27 pg/mL). When stratified by BMI, these intergroup differences reached significance among the participants with BMI ≥ 25 kg/m^2^ (*p* < 0.01), suggesting that adiposity may modulate the anti-inflammatory response to reformulated products.

These anti-inflammatory effects may stem both from the removal of potentially pro-inflammatory additives, such as carrageenan (E-407), which has been implicated in the disruption of the intestinal mucus layer and the dysregulation of tight junction proteins [53,54], and from the bioactivity of the natural extracts added during reformulation. Indeed, hydroxytyrosol has been shown to inhibit pro-inflammatory cytokine expression in LPS-stimulated human monocytes [55], and catechins modulate the NF-κB and MAPK signalling pathways in models of inflammatory bowel disease [56].

Finally, first-void urine nitrate concentrations declined significantly in the intervention group (80.0 ± 51.3 to 59.0 ± 23.0 µmol/L) while rising modestly in the control group (68.6 ± 50.7 to 74.6 ± 49.5 µmol/L), yielding a significant intergroup difference (*p* < 0.05). This pattern likely reflects the complete elimination of sodium nitrite (E-250) from the reformulated products, reducing endogenous nitrite and nitrate load. Given that ingested nitrites can react with nitrosating agents in the gut to form potentially carcinogenic N-nitrosamines [15], and considering EFSA’s concerns regarding these compounds in food [57], the substitution of synthetic nitrites with natural preservatives may enhance the safety profile of cooked meat products.

Minor shifts in microbial diversity likely reflect the compositional differences between the control and reformulated products, the latter being free of artificial additives and enriched with natural bioactive compounds. Previous studies have demonstrated that various food additives can alter gut microbiota composition and influence host health outcomes [56,57].

At the genus level, the most pronounced changes occurred in *Nitrospira* and *Nitrobacter*. Because *Nitrospira* is the only representative of the phylum Nitrospirota detected in our faecal samples, its significance mirrors that observed at the phylum level. In contrast, *Nitrobacter* abundance increased non-significantly in the control group (from 0.427 ± 0.587% to 1.053 ± 1.944%) but declined significantly in the intervention group (from 0.494 ± 0.845% to 0.163 ± 0.241%). A similar pattern was noted for *Rubrobacter*, which decreased in both groups but reached statistical significance only in the intervention arm. Additionally, *Candidatus Alysiosphaera* exhibited divergent trends: a reduction in the control group and an increase following consumption of the reformulated products. 

Bacteria belonging to the genus Nitrobacter and Nitrospira are nitrifying bacteria in-volved in nitrification by the oxidation of nitrite (NO_2_-) to nitrate (NO_3_-). Nitrobacter are a group of Gram-negative bacteria belonging to Proteobacteria, while Nitrospira form part of Nitrospirota [58,59]. These bacteria, which showed significant changes in this study, could play an important role in the pathway of nitrites and nitrates ingested in the diet. 

In the case of cooked meat products, the additive E-250 (sodium nitrite) is typically included to ensure their stability and organoleptic characteristics. The nitrites present in raw meat products can be reduced to nitrates, which are subsequently ingested upon consumption of the meat products in question [59]. Following ingestion, the nitrate-nitrite-nitric oxide pathway is initiated, with an initial conversion of nitrates to nitrites occurring in the oral cavity due to the action of commensal bacteria [60]. Subsequently, these nitrites enter the stomach lumen, where they give rise to nitric acid, which, through spontaneous degradation, generates nitric oxide and other nitrogen oxides. These compounds are responsible for the production of N-nitrosamines, which have been demonstrated to have adverse effects on human health following absorption in the intestine [61,62]. Furthermore, some of the ingested nitrates are not converted into nitrites; rather, they pass through the intestine and enter the bloodstream, where 75% are excreted in urine and the remainder is reabsorbed and concentrated in the salivary glands [63].

The relevant genera in this study, Nitrobacter and Nitrospira, may intervene in the pathway by acting on nitrites reaching the gut by oxidising them to nitrates, which would then pass into the bloodstream and be excreted in the urine, thereby preventing the for-mation of N-nitrosamines. Furthermore, an increased abundance of these bacteria is ob-served in the gut in conjunction with elevated nitrite levels. This may provide an explana-tion as to why higher levels of these bacteria were detected in the group that consumed the control products containing the E-250 additive. The group that consumed the products in which this additive was replaced by other natural ingredients exhibited a reduction in the levels of these microorganisms.

Despite these taxonomic shifts, the reformulation with natural extracts did not prevent the overall decline in faecal short-chain fatty acids (SCFAs) observed in both study arms. As SCFAs are primarily generated by microbial fermentation of dietary fibre, their decrease may be attributable to the low fibre content of the meat products and the concomitant replacement of other fibre-rich protein sources in the participants’ diets.

Across the three-hour postprandial period, VAS scores for the basal and control breakfasts remained essentially unchanged, whereas the reformulated breakfast consistently elicited significantly lower ratings of hunger, desire to eat, and prospective consumption and higher ratings of fullness. Thus, the reformulated meal demonstrated the greatest overall satiating effect.

As detailed in Table 10, the reformulated breakfast achieved the highest mean satiety scores, encompassing hunger suppression, perceived fullness, reduced desire to eat, and lower expected intake, with the control breakfast yielding intermediate results. Analysis of the areas under the curve (AUC) confirmed that the participants experienced significantly reduced hunger, desire to eat, and prospective consumption following the reformulated breakfast compared to both the basal and control meals (*p* < 0.05). Immediate satiety responses, expressed as the change in VAS from pre- to post-consumption, were similar across all three breakfasts, indicating equivalent initial satiation. However, the incremental VAS change at 180 min (ΔVAS_180_) revealed that the reformulated breakfast produced the most pronounced and sustained reduction in hunger three hours after ingestion (*p* < 0.05). These findings indicate that the reformulated breakfast not only matches other meals in inducing immediate fullness but also provides a more durable satiating effect over time.

To elucidate the mechanisms underlying the superior satiating effect of the reformulated breakfast, we first compared the macronutrient composition and digestibility profiles of the three meal variants. The in vitro analyses revealed that the reformulated cooked ham and turkey breast contain both higher total protein and significantly improved protein digestibility relative to the control products [27]. This finding is particularly relevant given that dietary protein is recognized as the most potent macronutrient for inducing satiety [64,65], although its efficacy is modulated by total protein load, source, and specific amino acid profile. Comparative studies indicate that animal-derived proteins generally elicit a greater suppression of appetite than plant-based proteins [27,66], yet pork, chicken and beef appear to generate equivalent satiety responses when matched for quantity [67].

Mechanistically, protein digestion stimulates the release of anorexigenic gut hormones, including cholecystokinin (CCK), glucagon-like peptide-1 (GLP-1), and peptide YY (PYY), while attenuating circulating levels of the orexigenic hormone ghrelin, collectively reducing hunger and prolonging fullness. Moreover, protein intake increases diet-induced thermogenesis, elevating postprandial energy expenditure and contributing further to the sensation of satiation [68,69]. High plasma concentrations of amino acids themselves act as metabolic signals in the hypothalamus to curb food intake [70], and bioactive peptides generated during gastric and small intestinal proteolysis potentiate enteroendocrine secretion of satiety mediators, including CCK, GLP-1, and serotonin [71].

Notably, reformulation enhanced the in vitro digestibility of leucine, a key branched-chain amino acid with recognized anorectic properties. In animal models, leucine supplementation suppresses food intake and body weight gain while improving glucose and lipid homeostasis via downregulation of hypothalamic AMPK and ACC phosphorylation (thereby lowering the AMP:ATP ratio) and upregulation of mTOR signalling [72,73]. Such data support the hypothesis that increasing both the quantity and the bioavailability of specific amino acids may favourably modulate satiety and, by extension, weight management [74].

In addition to protein optimization, the reformulated products were enriched with dietary fibre, the viscosity and fermentability of which prolong gastric emptying and intestinal transit, slow nutrient absorption, and enhance colonic production of short-chain fatty acids. These physiochemical actions on the gut lumen and mucosa stimulate the release of appetite-regulating peptides and correlate positively with satiety ratings obtained via visual analogue scales [75,76]. Collectively, these compositional and functional enhancements likely account for the markedly greater satiety and suppression of hunger observed following the consumption of the reformulated breakfast.

## 5. Limitations and Strengths of This Study

While the findings of this study offer valuable insights into the effects of reformulating processed meats to exclude synthetic additives and allergens, several limitations must be acknowledged that temper the scope and generalizability of our conclusions.

First, the intervention period was relatively short—just five weeks—which may be insufficient to capture long-term clinical outcomes or enduring shifts in microbiota composition. Dietary interventions often require extended durations to manifest measurable metabolic, inflammatory, or microbiome-related changes. Thus, future research should prioritize longer-term studies to better assess the durability and magnitude of physiological responses.

Second, the modest sample size and homogenous nature of our participant cohort—primarily healthy, young adults affiliated with a university setting—limit both the statistical power and external validity of this study. While this demographic offers controlled conditions for preliminary exploration, it does not reflect the broader population, particularly those with pre-existing health conditions, different dietary habits, or varied socio-demographic backgrounds. Future trials should strive for larger, more diverse samples to improve generalizability and enhance subgroup analyses.

Third, our dietary intake data relied on self-reported food records, which are inherently prone to recall bias, misreporting, and inter-individual variability. Though useful for feasibility in free-living studies, these limitations constrain the precision of nutrient exposure assessment. To address this, future protocols should consider the use of standardized pre-prepared meals and/or objective intake biomarkers (e.g., urinary nitrogen, alkylresorcinols, stable isotopes) to more accurately quantify dietary adherence and exposure.

In addition, this study lacked in-depth mechanistic analyses that might illuminate the biological underpinnings of the observed effects. While we reported changes in selected inflammatory markers, oxidative stress parameters, and microbiota composition, a more comprehensive understanding of the involved pathways would benefit from multi-omics approaches, such as transcriptomics, metabolomics, or metagenomics, and targeted profiling of microbiota-derived metabolites (e.g., bile acids, phenolic catabolites, short-chain fatty acids). Incorporating such tools in future research could help clarify causal links and identify molecular signatures associated with additive-free reformulations.

Despite these limitations, this study also presents several strengths. It employed a randomized, controlled design in a real-world dietary context, allowing for ecologically valid assessments of reformulated products without requiring extreme dietary changes. The intervention foods were carefully matched to their commercial counterparts in appearance, taste, and nutrient content (aside from additives), enhancing the internal validity of observed effects. Furthermore, the multi-dimensional assessment—including biochemical, microbiological, and behavioural measures—provides a broad perspective on the potential health implications of cleaner-label processed meats.

Together, these strengths position this study as a meaningful step towards understanding the health impact of reducing synthetic additives in processed foods, while its limitations offer clear direction for future, more robust investigations.

## 6. Conclusions

This research aimed to understand the reasons behind consumers’ rejection of additives and to explore the health effects of processed meat products free from additives and allergens. The additive perception survey revealed that consumers, in general, consider additive-free products as more natural and less toxic, where additives are rejected mainly by older people and those with less knowledge about them. To assess the actual health impacts of these products, a five-week clinical trial was conducted. The trial indicated that reformulation might influence certain inflammation markers, such as IL-1β and TNF-α. Additionally, it showed effects on nitrate regulation, with reduced nitrate excretion in urine and a decrease in nitrification-related bacteria in the gut microbiota. The natural antioxidants in the reformulated products demonstrated similar beneficial effects on oxidation markers (ox-LDL and GPx) as synthetic additives. Furthermore, the reformulated products showed a greater satiating effect, as measured by VAS scores. These findings suggest that the new reformulated meat products could appeal to consumers seeking more natural options and that such reformulations may offer notable health benefits.

## Figures and Tables

**Table 1:** Survey demographic data (N = 330).

Gender	
Male	128 (38.8%)
Female	192 (58.2%)
Prefer not to answer	10 (3.0%)
Age	
Generation Z (1998–2012)	185 (56.1%)
Millennials (1979–1997)	60 (18.2%)
Generation X (1968–1978)	40 (12.1%)
Baby Boomers (1946–1967)	45 (13.6%)
Allergenicity	
Allergic individuals	57 (17.3%)
Non-allergic individuals	273 (82.7%)
Education level	
No education/Primary school	8 (2.4%)
Secondary education	65 (19.7%)
University degree	179 (54.2%)
Master’s degree	59 (17.9%)
Doctor/Professor	19 (5.8%)

**Table 2:** Distribution of volunteer demographics.

	Control	Intervention	*p*-Value
Age (years)	26.6 ± 11.5	26.7 ± 11.7	0.97
Men	15	14	1.00
Women	14	15	
BMI (kg/m^2^)	23.7 ± 2.7	23.6 ± 2.8	0.98
Weight (kg)	66.6 ± 11.8	68.4 ± 12.7	0.58
Fat mass (%)	26.0 ± 9.0	26.9 ± 9.0	0.71
TC (mg/dL)	175.8 ± 34.6	178.0 ± 30.9	0.80
Ox- LDL (ng/mL)	238.6 ± 52.1	233.6 ± 52.4	0.71

*p*-values between the control and intervention groups were examined using Student’s *t*-test. The results are shown as mean ± standard deviation (SD) (*n* = 29).

**Table 3:** Nutritional values of breakfasts.

	Energy (KJ/kcal)	Protein (g)	Fat (g)	Carbohydrates (g)
Basal Breakfast	1548/370	7.4	11.5	59.1
Control Breakfast	1702/407	14.8	12	59.5
Reformulated Breakfast	1723/412	16.6	11.9	59.4

**Table 4:** Perception of food additives.

Questions	Number of Survey Respondents	% of Survey Respondents
What preference would you have for two products of similar taste and price that differ only in the presence of additives?		
I would choose the product without additives	235	71.2
I would choose the product with additives	9	2.7
I would be indifferent	86	26.1
Total	330	100.0
Do you consider a product to be more “natural” if it does not contain additives?		
Yes	259	78.2
No	72	21.8
Total	330	100.0
What do you think is the greatest benefit of an additive-free product?		
They are less toxic to me	151	45.8
They improve the sensory quality of the food	15	4.5
Improve intestinal health	52	15.8
They can help improve the immune system	23	7.0
They have no benefits	45	13.6
They are better digested	17	5.2
They are more nutritious	13	3.9
They help to care for the environment	14	4.2
Total	330	100.0
What is the biggest benefit you think a product with additives can have?		
They help to better preserve the food	203	61.5
Improve or preserve the nutritional value of the food	45	13.6
Improve the sensory quality of the food	40	12.1
They provide colour and change the taste	26	7.9
They give the food a consistent and smooth texture	4	1.2
They have no benefits	0	0
Total	330	100.0

**Table 5:** Question “What preference would you have for two products of similar taste and price that differ only in the presence of additives?”.

Demographics	I Would Choose the Product with Additives	I Would Choose the Product Without Additives	I Would Be Indifferent	Total	Sig.
Sex					
Male	2 (1.6%)	92 (71.9%)	34 (26.6%)	128 (100%)	χ^2^ = 1.157 df = 2 *p* < 0.561
Female	7 (3.6%)	138 (70.4%)	51 (26.0%)	196 (100%)	
Age group					
Generation Z (1998–2012)	5 (2.7%)	118 (63.8%)	62 (28.3%)	185 (100%)	χ^2^ = 20.03 df = 6 *p* < 0.003
Millennial (1979–1997)	2 (3.3%)	41 (68.3%)	17 (28.3%)	60 (100%)	
Generation X (1978–1968)	1 (2.5%)	36 (90%)	3 (7.5%)	40 (100%)	
Baby Boomer (1946–1967)	1 (2.2%)	40 (88.9%)	4 (8.9%)	45 (100%)	
Knowledge of additives					
Yes	5 (3.2%)	98 (62.4%)	54 (34.4%)	157 (100%)	χ^2^ = 11.463 df = 2 *p* < 0.03
No	4 (2.3%)	137 (79.2%)	32 (18.5%)	173 (100%)	

The significance of the perception of the respondents on food additives in terms of demographic data was tested using cross-tabulation and a chi-square test. The results are shown as the number of respondents and the percentage out of the total respondents. df: degree of freedom.

**Table 6:** Anthropometric measurements of the participants.

Measured Parameter	Group	Baseline	Final	*p*-Value (Time)	*p*-Value (Product × Time)
Weight (kg)	Control	66.6 ± 11.8	66.79 ± 11.8	0.95	1.00
	Intervention	68.4 ± 12.7	68.59 ± 13.2	0.95	
BMI (kg/m^2^)	Control	23.7 ± 2.7	23.7 ± 2.6	0.96	0.32
	Intervention	23.6 ± 2.8	23.7 ± 2.9	0.76	
Fat mass (%)	Control	26.0 ± 8.9	27.2 ± 8.5	0.62	0.66
	Intervention	26.9 ± 9.0	28.3 ± 9.4	0.57	
Abdominal circumference (cm)	Control	78.5 ± 8.0	78.2 ± 8.7	0.92	0.35
	Intervention	80.3 ± 10.2	79.2 ± 9.3	0.67	
WHR (cm waist–cm hip)	Control	0.79 ± 0.07	0.79 ± 0.08	0.98	0.82
	Intervention	0.80 ± 0.07	0.79 ± 0.06	0.35	

Significant intragroup (time) differences were determined using Student’s *t*-test. Significant differences between groups (product × time) were determined using ANOVA for repeated measures.

**Table 7:** Lipid, oxidative, and inflammatory markers before and after intervention.

	Group	Baseline	Final	*p*-Value (Time)	*p*-Value (Product × Time)
Lipid and glycaemic markers					
Basal glucose (mg/dL)	Control	83.2 ± 7.9	83.4 ± 8.2	0.94	0.77
	Intervention	82.7 ± 6.2	83.0 ± 6.3	0.83	
GOT (U/L)	Control	21.9 ± 8.8	22.4 ± 9.7	0.84	0.63
	Intervention	22.0 ± 10.3	20.3 ± 9.1	0.52	
GPT (U/L)	Control	22.8 ± 19.0	26.9 ± 19.9	0.43	0.17
	Intervention	18.6 ± 7.2	21.9 ± 7.7	0.09	
Triglycerides (mg/dL)	Control	70.9 ± 27.9	69.3 ± 37.4	0.86	0.22
	Intervention	75.0 ± 30.9	65.9 ± 29.5	0.26	
HDL cholesterol (mg/dL)	Control	65.4 ± 14.0	62.8 ± 13.8	0.49	0.90
	Intervention	66.9 ± 13.3	62.2 ± 12.7	0.17	
LDL cholesterol (mg/dL)	Control	98.6 ± 27.9	101.6 ± 33.5	0.71	0.87
	Intervention	100.5 ± 23.6	102.0 ± 28.0	0.83	
Total cholesterol (mg/dL)	Control	175.8 ± 34.6	180.1 ± 35.1	0.64	0.78
	Intervention	178.0 ± 30.9	182.8 ± 33.1	0.57	
Oxidation Markers					
Serum FRAP (μmol eq trolox/L)	Control	1045.7 ± 184.4	1091.2 ± 214.3	0.39	0.41
	Intervention	1032.6 ± 188.9	1052.4 ± 194.5	0.70	
Serum ABTS (μmol eq trolox/L)	Control	1228.7 ± 192.4	1219.6 ± 192.5	0.86	0.30
	Intervention	1175.3 ± 184.2	1146.0 ± 194.3	0.56	
Serum MDA (nmol/L)	Control	677.7 ± 70.5	712.5 ± 100.9	0.13	0.22
	Intervention	682.4 ± 57.1	669.5 ± 83.5	0.49	
Serum glutathione peroxidase (U/L)	Control	491.6 ± 382.9	340.8 ± 76.9	0.04	0.73
	Intervention	436.5 ± 172.0	311.3 ± 59.6	<0.01	
Serum catalase (U/mL)	Control	0.103 ± 0.018	0.104 ± 0.023	0.73	0.32
	Intervention	0.105 ± 0.015	0.102 ± 0.015	0.49	
Serum ox-LDL (ng/mL)	Control	238.6 ± 52.1	193.0 ± 40.2	<0.01	0.68
	Intervention	233.6 ± 52.4	192.4 ± 29.9	<0.01	
Inflammatory Markers					
Serum hs-CRP (mg/dL)	Control	3.20 ± 3.06	2.82 ± 2.54	0.61	0.55
	Intervention	2.02 ± 1.54	2.40 ± 1.63	0.37	
Serum TNF-α (pg/mL)	Control	18.28 ± 4.94	17.82 ± 4.40	0.71	0.84
	Intervention	17.13 ± 7.41	16.48 ± 5.98	0.72	
Serum TNF-α (pg/mL) IMC < 25	Control (n = 20)	19.80 ± 4.63	19.02 ± 3.94	0.58	0.47
	Intervention (n = 22)	17.31 ± 8.28	17.24 ± 6.57	0.98	
Serum TNF-α (pg/mL) IMC ≥ 25	Control (n = 9)	16.36 ± 5.44	16.94 ± 4.27	0.80	0.05
	Intervention (n = 7)	16.56 ± 3.97	14.08 ± 2.66	0.19	
Serum IL-1β (pg/mL)	Control	3.27 ± 1.64	4.68 ± 3.68	0.64	0.04
	Intervention	3.96 ± 2.74	4.01 ± 2.13	0.94	
Serum IL-1β (pg/mL) IMC < 25	Control (n = 20)	3.18 ± 1.86	4.87 ± 4.47	0.14	0.11
	Intervention (n = 22)	4.24 ± 2.74	4.41 ± 1.98	0.83	
Serum IL-1β (pg/mL) IMC ≥ 25	Control (n = 9)	3.28 ± 1.40	4.40 ± 1.41	0.11	<0.01
	Intervention (n = 7)	3.07 ± 2.75	2.77 ± 2.23	0.83	
Serum IL-6 (pg/mL)	Control	6.28 ± 9.48	6.19 ± 9.15	0.97	0.37
	Intervention	13.81 ± 24.61	12.26 ± 22.59	0.80	
Serum IL-10 (pg/mL)	Control	17.96 ± 32.35	22.14 ± 27.74	0.63	0.72
	Intervention	47.10 ± 99.20	48.40 ± 99.43	0.96	
Additive exposure markers					
Urinary nitrates (mg/L)	Control	68.6 ± 50.7	74.6 ± 49.5	0.65	**0.05 **
	Intervention	80.0 ± 51.3	59.0 ± 23.0	**0.05**	

Significant intragroup (time) differences were determined using Student’s *t*-test. Significant differences between groups (product × time) were determined using ANOVA for repeated measures. Significant values (*p* < 0.05) are expressed in bold.

**Table 8:** Impacted microbial features at the phylo and genus levels.

		Control			Intervention			Sig. (Time × Group) <0.05
		Baseline	Final	Sig. (Time) <0.05	Baseline	Final	Sig. (Time) <0.05	
Phylo								
Acidobacteriota		1.940 ± 1.607	2.201 ± 1.612		1.621 ± 1.794	2.948 ± 2.740	**0.033**	
Nitrospirota		0.877 ± 0.859	2.197 ± 2.922	**0.023**	1.135 ± 1.213	1.313 ± 1.478		**0.043**
Genus								
*Rubrobacter*		1.598 ± 1.641	1.294 ± 0.871		2.578 >± 2.771	1.422 ± 1.319	**0.047**	
*Nitrospira*		0.877 ± 0.859	2.197 ± 2.922	**0.023**	1.135 ± 1.213	1.313 ± 1.478		**0.043**
*Nitrobacter*		0.427 ± 0.587	1.053 ± 1.944		0.494 ± 0.845	0.163 ± 0.241	**0.047**	**0.010**
*Candidatus Alysiosphaera*		0.295 ± 0.389	0.222 ± 0.222		0.403 ± 0.478	0.798 ± 0.988		**0.024**

*p*-values between the standard and reformulated products were examined using a one-way analysis of variance (Tukey’s test). Bacteria with significant *p* in both meat matrices are presented in bold. The results are shown as mean percentage of relative abundance ± SD. Sig: significance.

**Table 9:** Faecal markers before and after intervention.

	Group	Baseline	Final	*p*-Value (Time)	*p*-Value (Product × Time)
FRAP in faeces (mmol eq Trolox/kg of faeces)	Control	150.9 ± 81.8	162.5 ± 107.6	0.648	0.938
	Intervention	160.7 ± 103.9	156.6 ± 109.9	0.884	
Acetic acid in faeces (mmol/kg of faeces)	Control	67.97 ± 43.51	51.55 ± 25.89	0.086	0.906
	Intervention	70.43 ± 45.84	55.29 ± 31.31	0.148	
Propionic acid in faeces (mmol/kg of faeces)	Control	76.27 ± 60.27	58.32 ± 47.07	0.211	0.613
	Intervention	69.37 ± 65.91	57.95 ± 55.39	0.476	
Butyric acid in faeces (mmol/kg of faeces)	Control	4.16 ± 6.12	4.15 ± 7.53	1.000	0.836
	Intervention	3.54 ± 6.52	3.27 ± 6.77	0.877	
Total short-chain fatty acids (mmol/kg of faeces)	Control	148.39 ± 76.31	114.03 ± 61.36	0.064	0.685
	Intervention	143.34 ± 78.19	116.52 ± 56.39	0.140	

Significant intragroup (time) differences were determined using Student’s *t*-test. Significant differences between groups (Product × Time) were determined using ANOVA for repeated measures. Significant values (*p* < 0.05) are expressed in bold.

**Table 10:** Visual analogue scale (VAS) results expressed as AUC, initial VAS, and ∆VAS_180_ score for each analysed breakfast.

	Basal Breakfast	Control Breakfast	Reformulated Breakfast
Area under curve (AUC)			
Hunger	766.18 ± 282.13 ^b^	681.82 ± 260.04 ^ab^	533.25 ± 233.95 ^a^
Fullness	712.39 ± 303.96 ^a^	721.84 ± 323.25 ^a^	813.43 ± 421.38 ^a^
Desire to eat	825.86 ± 342.91 ^b^	731.64 ± 298.24 ^ab^	557.57 ± 276.06 ^a^
Prospective food consumption	855.96 ± 297.70 ^b^	760.92 ± 260.23 ^ab^	576.11 ± 294.40 ^a^
Initial VAS (cm)			
Hunger	−3.36 ± 2.15 ^a^	−4.46 ± 2.37 ^a^	−4.37 ± 3.23 ^a^
Fullness	4.23 ± 2.99 ^a^	5.90 ± 2.03 ^a^	5.74 ± 3.05 ^a^
Desire to eat	−3.09 ± 3.44 ^a^	−2.64 ± 2.90 ^a^	−3.74 ± 3.10 ^a^
Prospective food consumption	−2.74 ± 2.59 ^a^	−3.22 ± 2.26 ^a^	−4.20 ± 2.21 ^a^
Incremental VAS180 score (cm)			
Hunger	4.28 ± 2.88 ^ab^	5.08 ± 2.47 ^b^	3.27 ± 2.97 ^a^
Fullness	−3.70 ± 2.89 ^a^	−5.36 ± 2.43 ^a^	−4.17 ± 2.73 ^a^
Desire to eat	3.55 ± 3.39 ^a^	3.02 ± 3.45 ^a^	2.53 ± 3.96 ^a^
Prospective food consumption	3.04 ± 3.08 ^a^	3.15 ± 3.03 ^a^	2.54 ± 3.18 ^a^

^a,b^ Different letters in superscript within the same column indicate statistically significant differences (*p* ≤ 0.05). The results are shown as mean ± SD (n = 21).

## Data Availability

The data presented in this study are available on request from the corresponding author. The data are not publicly available due to the presence of personal information from human participants and the related ethical restrictions.

## Supplementary Materials

The following supporting information can be downloaded at https://www.mdpi.com/article/10.3390/nu17101616/s1: File S1: Additives Perception Survey; File S2: CONSORT 2010 Checklist; File S3: Satiety Evaluation.

## References

[1] Abraham J., A I. (2020). Anxieties, Concerns and Facts about Meat Consumption and Health: A Short Review. J. Food Anim. Sci..

[2] Pereira P.M.d.C.C., Vicente A.F.d.R.B. (2013). Meat Nutritional Composition and Nutritive Role in the Human Diet. Meat Sci..

[3] Leroy F., Smith N.W., Adesogan A.T., Beal T., Iannotti L., Moughan P.J., Mann N. (2023). The Role of Meat in the Human Diet: Evolutionary Aspects and Nutritional Value. Anim. Front..

[4] González N., Marquès M., Nadal M., Domingo J.L. (2020). Meat Consumption: Which Are the Current Global Risks? A Review of Recent (2010–2020) Evidences. Food Res. Int..

[5] Harguess J.M., Crespo N.C., Hong M.Y. (2020). Strategies to Reduce Meat Consumption: A Systematic Literature Review of Experimental Studies. Appetite.

[6] Kumar N., Singh A., Sharma D.K., Kishore K. (2019). Toxicity of Food Additives. Food Safety and Human Health.

[7] Jiang J., Xiong Y.L. (2016). Natural Antioxidants as Food and Feed Additives to Promote Health Benefits and Quality of Meat Products: A Review. Meat Sci..

[8] Laganà P., Avventuroso E., Romano G., Gioffré M.E., Patanè P., Parisi S., Moscato U., Delia S. (2017). Use and Overuse of Food Additives in Edible Products: Health Consequences for Consumers. Chemistry and Hygiene of Food Additives.

[9] Younes M., Aquilina G., Castle L., Engel K.H., Fowler P., Frutos Fernandez M.J., Fürst P., Gürtler R., Husøy T., Mennes W. (2019). Re-Evaluation of Phosphoric Acid–Phosphates—Di-, Tri- and Polyphosphates (E 338–341, E 343, E 450–452) as Food Additives and the Safety of Proposed Extension of Use. EFSA J..

[10] Silva M.M., Lidon F.C. (2016). An Overview on Applications and Side Effects of Antioxidant Food Additives. Emir. J. Food Agric..

[11] Wu W., Zhou J., Xuan R., Chen J., Han H., Liu J., Niu T., Chen H., Wang F. (2022). Dietary κ-Carrageenan Facilitates Gut Microbiota-Mediated Intestinal Inflammation. Carbohydr. Polym..

[12] Munyaka P.M., Sepehri S., Ghia J.E., Khafipour E. (2016). Carrageenan Gum and Adherent Invasive Escherichia Coli in a Piglet Model of Inflammatory Bowel Disease: Impact on Intestinal Mucosa-Associated Microbiota. Front. Microbiol..

[13] Tobacman J.K. (2001). Review of Harmful Gastrointestinal Effects of Carrageenan in Animal Experiments. Environ. Health Perspect..

[14] Guimaraes D.A., Batista R.I.M., Tanus-Santos J.E. (2021). Nitrate and Nitrite-Based Therapy to Attenuate Cardiovascular Remodelling in Arterial Hypertension. Basic. Clin. Pharmacol. Toxicol..

[15] Deveci G., Tek N.A. (2023). N-Nitrosamines: A Potential Hazard in Processed Meat Products. J. Sci. Food Agric..

[16] Dusemund B., Gilbert J., Gott D., Kenigswald H., König J., Lambré C., Leblanc J.C., Mortensen A., Tobback P. (2012). Food Additives and Nutrient Sources Added to Food: Developments since the Creation of EFSA. EFSA J..

[17] Kwon Y., López-García R., Socolovsky S., Magnuson B. (2023). Global Regulations for the Use of Food Additives and Processing Aids. Present Knowledge in Food Safety: A Risk-Based Approach Through the Food Chain.

[18] Teixeira A., Rodrigues S. (2021). Consumer Perceptions towards Healthier Meat Products. Curr. Opin. Food Sci..

[19] Román S., Sánchez-Siles L.M., Siegrist M. (2017). The Importance of Food Naturalness for Consumers: Results of a Systematic Review. Trends Food Sci. Technol..

[20] Szücs V., Guerrero L., Claret A., Tarcea M., Szabó E., Bánáti D. (2014). Food Additives and Consumer Preferences: A Cross-Cultural Choice-Based Conjoint Analysis. Acta Aliment..

[21] Gökce A., Bozkir C., Seyitoglu D., Pehlivan E., Ozer A. (2017). Level of Food Additive Knowledge and Perceptions of Food Safety of University Students. Eur. J. Public Health.

[22] Martínez-Zamora L., Peñalver R., Ros G., Nieto G. (2021). Substitution of Synthetic Nitrates and Antioxidants by Spices, Fruits and Vegetables in Clean Label Spanish Chorizo. Food Res. Int..

[23] Powell M.J., Sebranek J.G., Prusa K.J., Tarté R. (2019). Evaluation of Citrus Fiber as a Natural Replacer of Sodium Phosphate in Alternatively-Cured All-Pork Bologna Sausage. Meat Sci..

[24] Martínez-Zamora L., Ros G., Nieto G. (2020). Synthetic vs. Natural Hydroxytyrosol for Clean Label Lamb Burgers. Antioxidants.

[25] Møller P. (2015). Satisfaction, Satiation and Food Behaviour. Curr. Opin. Food Sci..

[26] Stribiţcaia E., Evans C.E.L., Gibbons C., Blundell J., Sarkar A. (2020). Food Texture Influences on Satiety: Systematic Review and Meta-Analysis. Sci. Rep..

[27] Tremblay A., Bellisle F. (2015). Nutrients, Satiety, and Control of Energy Intake. Appl. Physiol. Nutr. Metab..

[28] Ayuso P., Quizhpe J., Yepes F., Miranzo D., Avellaneda A., Nieto G., Ros G. (2024). Improving the Nutritional Quality of Protein and Microbiota Effects in Additive- and Allergen-Free Cooked Meat Products. Foods.

[29] Marfell-Jones M., Olds T., Stewart A., Carter L. (2012). International Standards for Anthropometric Assessment.

[30] Slaughter M.R., O’brien P.J. (2000). Fully-Automated Spectrophotometric Method for Measurement of Antioxidant Activity of Catalase. Clin. Biochem..

[31] Benzie I.F.F., Strain J.J. (1996). The Ferric Reducing Ability of Plasma (FRAP) as a Measure of “‘Antioxidant Power’”: The FRAP Assay. Anal. Biochem..

[32] Tvarijonaviciute A., Aznar-Cayuela C., Rubio C.P., Ceron J.J., López-Jornet P. (2017). Evaluation of Salivary Oxidate Stress Biomarkers, Nitric Oxide and C-Reactive Protein in Patients with Oral Lichen Planus and Burning Mouth Syndrome. J. Oral. Pathol. Med..

[33] Aust S.D., Fleischer S. (1978). Microsomal Lipid Peroxidation. Biomembranes—Part C: Biological Oxidations.

[34] Yang X., Sun W., Hou D., Wang T., Li C., Luo Y., Zhang S., Shen L., Liu W., Wu D. (2021). The Degree of Plasma Oxidized Low-Density Lipoprotein Level Decrease Is Related to Clinical Outcomes for Patients with Acute Ischemic Stroke. Dis. Markers.

[35] Panzella L., Pérez-Burillo S., Pastoriza S., Martín M.Á., Cerruti P., Goya L., Ramos S., Rufián-Henares J.Á., Napolitano A., d’Ischia M. (2017). High Antioxidant Action and Prebiotic Activity of Hydrolyzed Spent Coffee Grounds (HSCG) in a Simulated Digestion–Fermentation Model: Toward the Development of a Novel Food Supplement. J. Agric. Food Chem..

[36] Gołębiewski M., Tretyn A. (2020). Generating Amplicon Reads for Microbial Community Assessment with Next-generation Sequencing. J. Appl. Microbiol..

[37] Callahan B.J., McMurdie P.J., Rosen M.J., Han A.W., Johnson A.J.A., Holmes S.P. (2016). DADA2: High-Resolution Sample Inference from Illumina Amplicon Data. Nat. Methods.

[38] Murali A., Bhargava A., Wright E.S. (2018). IDTAXA: A Novel Approach for Accurate Taxonomic Classification of Microbiome Sequences. Microbiome.

[39] McMurdie P.J., Holmes S. (2013). Phyloseq: An R Package for Reproducible Interactive Analysis and Graphics of Microbiome Census Data. PLoS ONE.

[40] Lozupone C., Knight R. (2005). UniFrac: A New Phylogenetic Method for Comparing Microbial Communities. Appl. Environ. Microbiol..

[41] Flint A., Raben A., Blundell J.E., Astrup A. (2000). Reproducibility, Power and Validity of Visual Analogue Scales in Assessment of Appetite Sensations in Single Test Meal Studies. Int. J. Obes. Relat. Metab. Disord..

[42] He J., Votruba S., Venti C., Krakoff J. (2011). Higher Incremental Insulin Area under the Curve during Oral Glucose Tolerance Test Predicts Less Food Intake and Weight Gain. Int. J. Obes..

[43] Shim S.M., Seo S.H., Lee Y., Moon G.I., Kim M.S., Park J.H. (2011). Consumers’ Knowledge and Safety Perceptions of Food Additives: Evaluation on the Effectiveness of Transmitting Information on Preservatives. Food Control.

[44] Buchler S., Smith K., Lawrence G. (2010). Food Risks, Old and New: Demographic Characteristics and Perceptions of Food Additives, Regulation and Contamination in Australia. J. Sociol..

[45] WHO (2011). Waist Circumference and Waist–Hip Ratio. WHO Expert..

[46] Khodayari S., Sadeghi O., Safabakhsh M., Mozaffari-Khosravi H. (2022). Meat Consumption and the Risk of General and Central Obesity: The Shahedieh Study. BMC Res. Notes.

[47] Wang Y., Beydoun M.A. (2009). Meat Consumption Is Associated with Obesity and Central Obesity among US Adults. Int. J. Obes..

[48] Poznyak A.V., Nikiforov N.G., Markin A.M., Kashirskikh D.A., Myasoedova V.A., Gerasimova E.V., Orekhov A.N. (2021). Overview of OxLDL and Its Impact on Cardiovascular Health: Focus on Atherosclerosis. Front. Pharmacol..

[49] Lang Y., Gao N., Zang Z., Meng X., Lin Y., Yang S., Yang Y., Jin Z., Li B. (2024). Classification and Antioxidant Assays of Polyphenols: A Review. J. Future Foods.

[50] Lubos E., Loscalzo J., Handy D.E. (2011). Glutathione Peroxidase-1 in Health and Disease: From Molecular Mechanisms to Therapeutic Opportunities. Antioxid. Redox Signal.

[51] Kaneko N., Kurata M., Yamamoto T., Morikawa S., Masumoto J. (2019). The Role of Interleukin-1 in General Pathology. Inflamm. Regen..

[52] D’Esposito V., Di Tolla M.F., Lecce M., Cavalli F., Libutti M., Misso S., Cabaro S., Ambrosio M.R., Parascandolo A., Covelli B. (2022). Lifestyle and Dietary Habits Affect Plasma Levels of Specific Cytokines in Healthy Subjects. Front. Nutr..

[53] Borsani B., De Santis R., Perico V., Penagini F., Pendezza E., Dilillo D., Bosetti A., Zuccotti G.V., D’auria E. (2021). The Role of Carrageenan in Inflammatory Bowel Diseases and Allergic Reactions: Where Do We Stand?. Nutrients.

[54] Martino J.V., Van Limbergen J., Cahill L.E. (2017). The Role of Carrageenan and Carboxymethylcellulose in the Development of Intestinal Inflammation. Front. Pediatr..

[55] Zhang X., Cao J., Zhong L. (2009). Hydroxytyrosol Inhibits Pro-Inflammatory Cytokines, INOS, and COX-2 Expression in Human Monocytic Cells. Naunyn Schmiedebergs Arch. Pharmacol..

[56] Fan F.-Y., Sang L.-X., Jiang M. (2017). Catechins and Their Therapeutic Benefits to Inflammatory Bowel Disease. Molecules.

[57] Schrenk D., Bignami M., Bodin L., Chipman J.K., del Mazo J., Hogstrand C., Hoogenboom L., Leblanc J.C., Nebbia C.S., Nielsen E. (2023). Risk Assessment of N-Nitrosamines in Food. EFSA J..

[58] Cao Y., Liu H., Qin N., Ren X., Zhu B., Xia X. (2020). Impact of Food Additives on the Composition and Function of Gut Microbiota: A Review. Trends Food Sci. Technol..

[59] Roca-Saavedra P., Mendez-Vilabrille V., Miranda J.M., Nebot C., Cardelle-Cobas A., Franco C.M., Cepeda A. (2017). Food Additives, Contaminants and Other Minor Components: Effects on Human Gut Microbiota—A Review. J. Physiol. Biochem..

[60] Grundmann G.L., Neyra M., Normand P. (2000). High-Resolution Phylogenetic Analysis of NO_2_/–Oxidizing Nitrobacter Species Using the Rrs-Rrl IGS Sequence and Rrl Genes. Int. J. Syst. Evol. Microbiol..

[61] Vijayan A., Vattiringal Jayadradhan R.K., Pillai D., Prasannan Geetha P., Joseph V., Isaac Sarojini B.S. (2021). Nitrospira as Versatile Nitrifiers: Taxonomy, Ecophysiology, Genome Characteristics, Growth, and Metabolic Diversity. J. Basic. Microbiol..

[62] Honikel K.O. (2008). The Use and Control of Nitrate and Nitrite for the Processing of Meat Products. Meat Sci..

[63] Karwowska M., Kononiuk A. (2020). Nitrates/Nitrites in Food-Risk for Nitrosative Stress and Benefits. Antioxidants.

[64] Weitzberg E., Lundberg J.O. (2013). Novel Aspects of Dietary Nitrate and Human Health. Annu. Rev. Nutr..

[65] Shi J., Zhao D., Song S., Zhang M., Zamaratskaia G., Xu X., Zhou G., Li C. (2020). High-Meat-Protein High-Fat Diet Induced Dysbiosis of Gut Microbiota and Tryptophan Metabolism in Wistar Rats. J. Agric. Food Chem..

[66] Van Hul M., Cani P.D. (2023). The Gut Microbiota in Obesity and Weight Management: Microbes as Friends or Foe?. Nat. Rev. Endocrinol..

[67] Morell P., Fiszman S. (2017). Revisiting the Role of Protein-Induced Satiation and Satiety. Food Hydrocoll..

[68] Bonnema A.L., Altschwager D., Thomas W., Slavin J.L. (2015). The Effects of a Beef-Based Meal Compared to a Calorie Matched Bean-Based Meal on Appetite and Food Intake. J. Food Sci..

[69] Nielsen L.V., Kristensen M.D., Klingenberg L., Ritz C., Belza A., Astrup A., Raben A. (2018). Protein from Meat or Vegetable Sources in Meals Matched for Fiber Content Has Similar Effects on Subjective Appetite Sensations and Energy Intake-A Randomized Acute Cross-Over Meal Test Study. Nutrients.

[70] Charlton K.E., Tapsell L.C., Batterham M.J., Thorne R., O’Shea J., Zhang Q., Beck E.J. (2011). Pork, Beef and Chicken Have Similar Effects on Acute Satiety and Hormonal Markers of Appetite. Appetite.

[71] Veldhorst M., Smeets A., Soenen S., Hochstenbach-Waelen A., Hursel R., Diepvens K., Lejeune M., Luscombe-Marsh N., Westerterp-Plantenga M. (2008). Protein-Induced Satiety: Effects and Mechanisms of Different Proteins. Physiol. Behav..

[72] Muliadi R.D., Kartawidjajaputra F., Antono L. (2022). The Effect of High Protein Milk Supplementation on Satiety in Normal Weight Subjects. Food Res..

[73] Joseph R.J., Alonso-Alonso M., Bond D.S., Pascual-Leone A., Blackburn G.L. (2011). The Neurocognitive Connection between Physical Activity and Eating Behavior. Obes. Rev..

[74] Santos-Hernández M., Miralles B., Amigo L., Recio I. (2018). Intestinal Signaling of Proteins and Digestion-Derived Products Relevant to Satiety. J. Agric. Food Chem..

[75] Zhang Y., Guo K., LeBlanc R.E., Loh D., Schwartz G.J., Yu Y.H. (2007). Increasing Dietary Leucine Intake Reduces Diet-Induced Obesity and Improves Glucose and Cholesterol Metabolism in Mice via Multimechanisms. Diabetes.

[76] Clark M.J., Slavin J.L. (2013). The Effect of Fiber on Satiety and Food Intake: A Systematic Review. J. Am. Coll. Nutr..

