# Supplementary material for: Exploring the Health Effects of New Additive- and Allergen-Free Reformulated Cooked Meat Products: Consumer Survey, Clinical Trial, and Perceived Satiety
Source: Nutrients. 2025 May 8;17(10):1616. doi: 10.3390/nu17101616 (PMC12114518; doi:10.3390/nu17101616)
Supplement: Supplementary file 1 [file nutrients-17-01616-s001.zip › Supplementary Material S1 Additives Perception Survey final.pdf]

Welcome and Thank You for Participating!

Thank you for taking part in this survey on **ADDITIVES IN COOKED PROCESSED MEAT PRODUCTS AND THEIR CONSUMPTION**.

The aim of this survey is to gather anonymous information from the general public—particularly university students and staff—regarding the habits that influence the consumption of certain meat products and the role that additives play in product selection.

The insights gathered will support further research and contribute to the development of food options tailored to the population’s individual nutritional needs.

If you have any questions, please feel free to contact us at: **meatcleanlabel@um.es**

The Research Team

Survey on perception of food additives and their relation to health

Respondent demographics

1. Age

- ◯ Under 18 years of age.
- ◯ 18 to 24 years old.
- ◯ 25 to 34 years old.
- ◯ 35 to 44 years old.
- ◯ 45 to 54 years old.
- ◯ Older than 55 years old.

2. Sex

◯ Man ◯ Woman

3. Weight

- ◯ Less than 45 kg.
- ◯ 45-55 kg.
- ◯ 55-65 kg.
- ◯ 65-75 kg.
- ◯ 75-85 kg.
- ◯ 85-95 kg.
- ◯ More than 95 kg.

4. Height

- ◯ Less than 150 cm.
- ◯ From 160 to 170 cm.
- ◯

- ○ From 170 to 180 cm.
- ○ From 180 to 190 cm.
- ○ Over 190 cm.

5. Level of education

- ○ No primary education/studies (compulsory secondary education).
- ○ Secondary education (Baccalaureate/Vocational Training/Non-university degree).
- ○ Undergraduate student.
- ○ Master's student.
- ○ Non-university graduate.
- ○ University graduate/graduate
- ○ Doctor/Teacher.

General issues on food additives

6. Are you aware of food additives?

○ Yes ○ No

7. E-200 additives are:

- ○ Colouring agents.
- ○ Preservatives.
- ○ Acidity regulators.
- ○ Antioxidants.

Product choice aspects.

8. On a scale of 1 to 10, how important do you think the health benefits of a product are to your decision to buy it?

	1	2	3	4	5	6	7	8	9	10	
None	○	○	○	○	○	○	○	○	○	○	A lot

9. **What preference would you have for two products of similar taste and price that differ only in the presence of additives?**

- ◯ I would choose the product with additives.
- ◯ I would choose the product without additives.
- ◯ I would be indifferent.

10. **Do you consider a product to be more "natural" if it does not contain additives?**

- ◯ Yes
- ◯ No

10. **En On a scale of 1 to 10, how important do you think it is for a product to be "100% natural" when you buy it?**

	1	2	3	4	5	6	7	8	9	10	
None	◯	◯	◯	◯	◯	◯	◯	◯	◯	◯	A lot

11. **On a scale of 1 to 10, how important do you think it is that a product does not contain any additives when you buy it?**

	1	2	3	4	5	6	7	8	9	10	
None	◯	◯	◯	◯	◯	◯	◯	◯	◯	◯	A lot

12. **On a scale of 1 to 10, how much health benefit do you think there would be if a food did not contain any additives compared to one that did?**

	1	2	3	4	5	6	7	8	9	10	
No benefit	◯	◯	◯	◯	◯	◯	◯	◯	◯	◯	A lot of benefit

13. What do you think is the biggest benefit of an additive-free product?

- ◯ They are better digested.
- ◯ They can help improve the immune system.
- ◯ They are less toxic to me.
- ◯ They improve the sensory quality of the food.
- ◯ They help to care for the environment.
- ◯ They are more nutritious.
- ◯ Improve intestinal health.
- ◯ They have no benefits.

14. What do you think is the greatest benefit of a product with additives?

- ◯ They improve or preserve the nutritional value of the food.
- ◯ They give the food a consistent and smooth texture.
- ◯ They provide colour and change the taste.
- ◯ Mejoran la calidad sensorial del alimento.
- ◯ They help to better preserve the food.
- ◯ They have no benefits.

15. How important to your health do you consider the following claims when buying a product?

Reduced salt content:

	1	2	3	4	5	6	7	8	9	10	
None	◯	◯	◯	◯	◯	◯	◯	◯	◯	◯	A lot

Reduced fat content:

	1	2	3	4	5	6	7	8	9	10	
None	○	○	○	○	○	○	○	○	○	○	A lot

High protein content:

	1	2	3	4	5	6	7	8	9	10	
None	○	○	○	○	○	○	○	○	○	○	A lot

Additive-free:

	1	2	3	4	5	6	7	8	9	10	
None	○	○	○	○	○	○	○	○	○	○	A lot

Allergen-free:

	1	2	3	4	5	6	7	8	9	10	
None	○	○	○	○	○	○	○	○	○	○	A lot

High antioxidant content:

	1	2	3	4	5	6	7	8	9	10	
None	○	○	○	○	○	○	○	○	○	○	A lot

High fibre content:

	1	2	3	4	5	6	7	8	9	10	
None	○	○	○	○	○	○	○	○	○	○	A lot

16. How often do you usually check the following elements of a product at the time of purchase?

Nutritional table

	1	2	3	4	5	6	7	8	9	10	
Never	◯	◯	◯	◯	◯	◯	◯	◯	◯	◯	Always

List of ingredients.

	1	2	3	4	5	6	7	8	9	10	
Never	◯	◯	◯	◯	◯	◯	◯	◯	◯	◯	Always

17. Do you have any food intolerances or allergies? If yes, please specify:

◯ Yes ◯ No

18. On a scale of 1 to 10, how important do you think it is for a product to be allergen-free when you buy it?

	1	2	3	4	5	6	7	8	9	10	
None	◯	◯	◯	◯	◯	◯	◯	◯	◯	◯	A lot

19. What do you think is the biggest benefit of an allergen-free product for you?

◯ I digest them better.

◯ They can help improve the immune system.

◯ They are less toxic to me.

◯ They improve the sensory quality of the food.

They help to care for the environment.

- ◯
- ◯ I consider them more nutritious.
- ◯ Improve intestinal health.
- ◯ They are of no benefit to me.

Perception aspects of additives in meat products.

20. How often do you consume meat products per week?

- ◯ Never.
- ◯ 1-2 times a week.
- ◯ 3-4 times a week.
- ◯ 5-6 times a week.
- ◯ Every day.

21. How do you think meat can influence health?

	1	2	3	4	5	6	7	8	9	10	
Very negatively	◯	◯	◯	◯	◯	◯	◯	◯	◯	◯	Very positive

22. Of the health benefits of meat, which is the most important to you?

- ◯ They are a great source of vitamin B12.
- ◯ They are a high quality source of protein.
- ◯ They are a source of essential fatty acids.
- ◯ They provide minerals such as iron and zinc.
- ◯ I do not see any benefit.

23. Do you think any of the following negative effects may be associated with meat consumption?

- ◯ Increased risk of hypertension.
- ◯ Increased risk of diabetes.
- ◯ Increased blood cholesterol levels.
- ◯ Increased risk of becoming overweight.
- ◯ I do not consider them to be prejudiced.

24. What is your main concern in the area of health and nutrition?

- ◯ Overweight.
- ◯ Hypertension.
- ◯ High cholesterol.
- ◯ Diabetes.
- ◯ Alergies.
- ◯ Food intolerances.
- ◯ Gut health.

25. On a scale of 1 to 10, do you consider a meat product such as cooked ham to be healthy?

	1	2	3	4	5	6	7	8	9	10	Very healthy
Unhealthy	◯	◯	◯	◯	◯	◯	◯	◯	◯	◯	

26. On a scale of 1 to 10, how important do you think it is that a cooked ham does not contain any additives when you buy it?

None	1	2	3	4	5	6	7	8	9	10	A lot
	◯	◯	◯	◯	◯	◯	◯	◯	◯	◯	

27. On a scale of 1 to 10, how much health benefit do you think a cooked ham that does not contain any additives and allergens could have compared to a normal cooked ham?

	1	2	3	4	5	6	7	8	9	10	
No benefits	○	○	○	○	○	○	○	○	○	○	A lot of benefits
