# Supplementary material for: Exploring the Health Effects of New Additive- and Allergen-Free Reformulated Cooked Meat Products: Consumer Survey, Clinical Trial, and Perceived Satiety
Source: Nutrients. 2025 May 8;17(10):1616. doi: 10.3390/nu17101616 (PMC12114518; doi:10.3390/nu17101616)

CONSORT 2010 Checklist for Randomized Trials

Section/Topic	Item No.	Checklist Item	Reported on Page No.
**Title and Abstract**	1a	Identification as a randomized trial in the title	Title Page
	1b	Structured summary of trial design, methods, results, and conclusions	Abstract
**Introduction**	2a	Scientific background and explanation of rationale	Introduction
	2b	Specific objectives or hypotheses	Introduction
**Methods**	3a	Description of trial design (e.g., parallel, factorial) including allocation ratio	Methods – Trial Design
	3b	Important changes to methods after trial commencement, with reasons	Not Applicable
	4a	Eligibility criteria for participants	Methods – Participants
	4b	Settings and locations where the data were collected	Methods – Trial Design
	5	The interventions for each group with sufficient details to allow replication	Methods – Trial Design
	6a	Completely defined pre-specified primary and secondary outcome measures	Methods – Study Variables
	6b	Any changes to trial outcomes after the trial commenced, with reasons	Not Applicable
	7a	How sample size was determined	Methods – Statistical Analysis
	7b	When applicable, explanation of any interim analyses and stopping guidelines	Not Applicable
	8a	Method used to generate the random allocation sequence	Methods – Trial Design
	8b	Type of randomization; details of any restriction (e.g., blocking, stratification)	Methods – Trial Design
	9	Mechanism used to implement the random allocation sequence (e.g., sequentially numbered containers), describing any steps taken to conceal the sequence until interventions were assigned	Methods – Trial Design
	10	Who generated the random allocation sequence, who enrolled participants, and who assigned participants to interventions	Methods – Trial Design
	11a	If done, who was blinded after assignment to interventions and how	Methods – Trial Design
	11b	If relevant, description of the similarity of interventions	Methods – Trial Design

Section/Topic	Item No.	Checklist Item	Reported on Page No.
	12a	Statistical methods used to compare groups for primary and secondary outcomes	Methods – Statistical Analysis
	12b	Methods for additional analyses, such as subgroup analyses and adjusted analyses	Methods – Statistical Analysis
**Results**	13a	For each group, the numbers of participants who were randomly assigned, received intended treatment, and were analyzed for the primary outcome	Results – Participant Flow
	13b	For each group, losses and exclusions after randomization, together with reasons	Results – Participant Flow
	14a	Dates defining the periods of recruitment and follow-up	Methods – Trial Design
	14b	Why the trial ended or was stopped	Not Applicable
	15	A table showing baseline demographic and clinical characteristics for each group	Results – Baseline Characteristics
	16	For each group, number of participants (denominator) included in each analysis and whether the analysis was by original assigned groups	Results – Outcomes
	17a	For each primary and secondary outcome, results for each group, and the estimated effect size and its precision (e.g., 95% confidence interval)	Results – Outcomes
	17b	For binary outcomes, presentation of both absolute and relative effect sizes is recommended	Not Applicable
	18	Results of any other analyses performed, including subgroup analyses and adjusted analyses, distinguishing pre-specified from exploratory	Results – Additional Analyses
	19	All important harms or unintended effects in each group	Results – Harms
**Discussion**	20	Trial limitations, addressing sources of potential bias, imprecision, and, if relevant, multiplicity of analyses	Discussion
	21	Generalizability (external validity, applicability) of the trial findings	Discussion
	22	Interpretation consistent with results, balancing benefits and harms, and considering other relevant evidence	Discussion
**Other Information**	23	Registration number and name of trial registry	Methods – Trial Design
	24	Where the full trial protocol can be accessed, if available	Not Specified
	25	Sources of funding and other support (e.g., supply of drugs), role of funders	Acknowledgments

CONSORT 2010 Flow Diagram

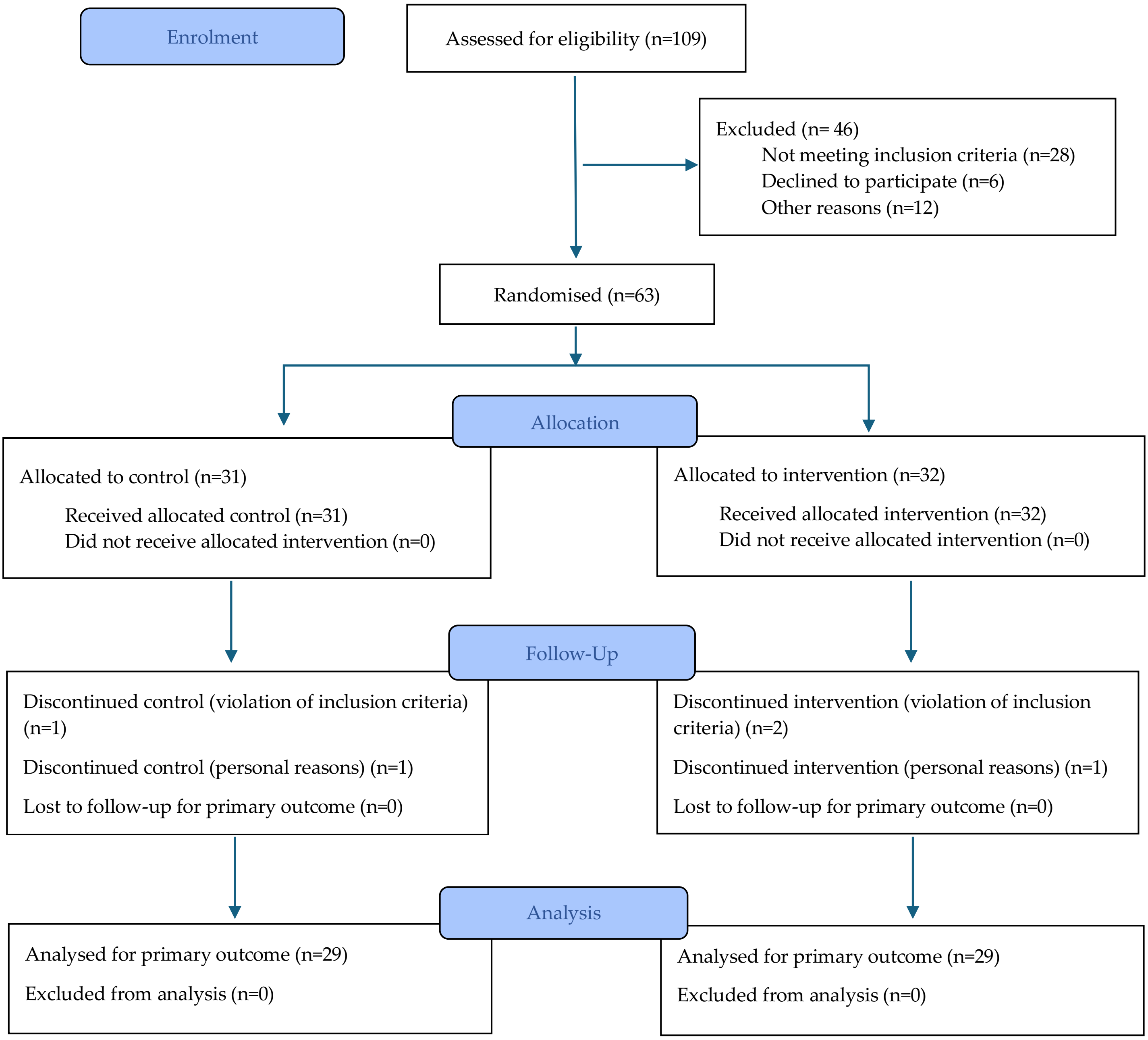

Supplement: Supplementary file 1 [file nutrients-17-01616-s001.zip › Supplementary Material S2- CONSORT 2010 Checklist and Flow Diagram.pdf]
