# Supplementary material for: Exploring the Health Effects of New Additive- and Allergen-Free Reformulated Cooked Meat Products: Consumer Survey, Clinical Trial, and Perceived Satiety
Source: Nutrients. 2025 May 8;17(10):1616. doi: 10.3390/nu17101616 (PMC12114518; doi:10.3390/nu17101616)
Supplement: Supplementary file 1 [file nutrients-17-01616-s001.zip › Supplementary Material S3 Satiety Evaluation.pdf]

INFORMED CONSENT DECLARATION

Mr/Ms.………
…………………….., aged ………. years, with ID number …………………….., declares that they have been informed about the potential benefits of participating in the research project entitled: **"RESEARCH AND DEVELOPMENT OF MINIMALLY PROCESSED AND HYPOALLERGENIC MEAT PRODUCTS (HIPOCARNE)"**, directed by **Professor Gaspar Ros Berruezo**, with the aim of determining the potential satiating effect of meat products without synthetic additives. This study has been approved by the Ethics Committee of the University of Murcia. Contact telephone: +34 968 364794 and email: gros@um.es.

I have been informed of the possible adverse effects that participation in this project could have on my well-being and health, having read the participant information sheet regarding the study in question.

I have been informed that my personal data will be processed with my consent for scientific research purposes by the University of Murcia. The data retention period will be the minimum necessary to ensure the completion of the study or project. Nevertheless, my identifying data, to ensure optimal privacy conditions and when the study procedure allows, may be anonymized or pseudonymized. In any case, any identifying information collected will be deleted when no longer necessary.

I have been informed that any query regarding the processing of my personal data in this study, or to request access, rectification, erasure, restriction, or objection, may be directed to: protecciondedatos@um.es. I have also been informed of my right to lodge a complaint with the Spanish Data Protection Agency.

I have been given a copy of the information sheet and a copy of this informed consent, dated and signed.

Taking all this into account, I hereby give my consent for this data and sample collection to take place and to be used to meet the specified objectives of the project.

Participant's Full Name

Participant's Signature

Date

Researcher's Signature: ____

INFORMATION SHEET

Palatability and satiety survey

Study Title:

"RESEARCH AND DEVELOPMENT OF MINIMALLY PROCESSED AND HYPOALLERGENIC MEAT PRODUCTS (HIPOCARNE): Design of new minimally processed, additive-free, and hypoallergenic protein foods."

What is the aim of the study?

The main aim of this study is to identify new natural ingredients capable of replacing the function of certain technological additives, for their application in the development of cleaner and more hypoallergenic protein formulations with scientifically proven benefits for consumer well-being.

One of the specific objectives is to analyze the potential satiating effect of additive- and allergen-free products through various questionnaires.

The study will take place in the sensory evaluation room located on the sixth floor of the Vitalys Building at the University of Murcia, Espinardo Campus. It will consist of three visits during which participants will consume three different breakfasts and complete brief surveys.

Why have I been invited to participate in this study?

You are invited to participate because you meet the criteria we are looking for: adults between the ages of 18 and 65, with a body mass index (BMI) between 18.5 and 29.9 kg/m^2.

What will I have to do if I decide to participate?

If you choose to participate, you will be asked to sign an informed consent form, since participation is voluntary and you may withdraw at any time without any consequence.

The study involves three visits, all of which require you to come in a fasting state. There will be at least one week between each visit. Each session will involve the following (as shown in the accompanying diagram): you will consume a breakfast (juice and two

toasts with olive oil, tomato, cooked ham, and turkey breast). You will then complete a series of short questionnaires about your sensations every 45 minutes over a period of three hours. You are not required to stay at the facility to complete these questionnaires.

In total, you will complete seven very brief questionnaires per session, each taking less than one minute.

During these three hours, you should not eat any food in order to avoid interfering with the results. However, you may drink water, provided you drink approximately the same amount on each of the three study days. It is also very important to remain relatively at rest during this time.

Important: On the day before each visit, you must not consume any alcohol, and you must abstain from eating or drinking anything (except water) from 11:59 PM until the time of your visit.

During the study, you should maintain your normal lifestyle. We understand that the three visits may interfere with your commitments and responsibilities, so we will be flexible and do our best to accommodate your availability.

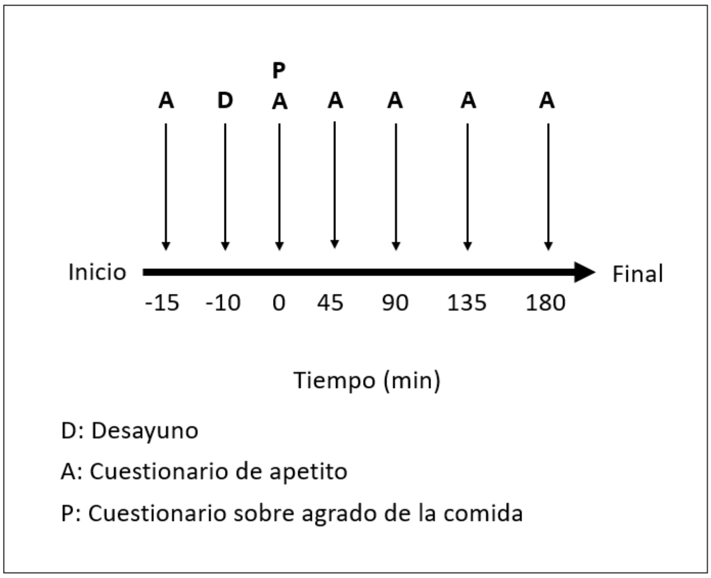

What are the possible risks or disadvantages of participating in the study?

All the foods provided are commonly available in supermarkets and have passed the required quality controls, so they pose no risk to your health.

What are the benefits of participating in the study?

Aside from the economic benefit of having three free breakfasts, we cannot guarantee any personal benefit from your participation. However, we can provide you with nutritional advice if you wish.

Furthermore, the knowledge gained from this study may provide valuable insights into the foods studied, meaning your participation contributes to the advancement of human nutrition science.

Methodology and tool for the surveys

Visual Analogue Scale (VAS) is a commonly used tool in surveys and studies to measure **subjective characteristics** or **sensations** that cannot be directly measured, such as **satiety**, **hunger**, **pain**, or **well-being**.

VAS Definition (Non-Quantified)

In this survey, the **Visual Analogue Scale (VAS)** is presented as a **horizontal line without numerical values**, anchored by **descriptive phrases at each end**. Participants are asked to place a **mark (☓ or X)** on the line to reflect their current sensation.

This format captures subjective feelings (e.g., hunger or fullness) without requiring the participant to interpret or assign a specific number.

Example (Non-Quantified VAS – Hunger)

How hungry do you feel right now?

Not at all hungry   Extremely hungry
|--|
☓

The position of the mark will be **measured by the research team**, to convert it into a score for analysis — **but the participant never sees numbers**. We believe that this method is easier and more intuitive for participants, reduces bias from numbers or midpoint cues, allows for fine-grained subjective reporting.

Who can I contact for more information?

If you want more information or need clarification about the study, you can contact the following people, who will be happy to assist you:

- Email: pablo.ayuson@um.es and jhazminedith.quizhper@um.es
- Pablo Ayuso Nicolás (Phone: +34 640 XXXX89)

- Jhazmin Edith Quizhpe Romero (Phone: +34 629 XXXX09)

Once again, thank you for taking the time to read all the information included in this document.

Welcome and Thank You for Participating!

Thank you for taking part in this survey on **Palatability and satiety surveis**.

The aim of this survey is to gather anonymous information from the general public—particularly university students and staff—regarding the habits that influence the consumption of certain meat products and the role that additives play in product selection.

The insights gathered will support further research and contribute to the development of food options tailored to the population's individual nutritional needs.

If you have any questions, please feel free to contact us at: **meatcleanlabel@um.es**

The Research Team

Palatability survey

INSTRUCTIONS FOR PARTICIPANTS:

Please read each question carefully and place a mark on the line at the point that best represents your experience with the meal you have just consumed.

PLEASE ANSWER THE FOLLOWING QUESTIONS:

1. How PLEASANT was this meal?

Not at all pleasant *Extremely pleasant*

|--|

2. How much would you LIKE TO EAT more of this meal?

Not at all *Very much*

|--|

3. How TASTY was this meal?

Not at all tasty *Very tasty*

|--|

4. How SWEET was this meal?

Not at all sweet *Very sweet*

|---|

5. How SALTY was this meal?

Not at all salty *Very salty*

|---|

6. How PLEASANT was the FLAVOR of this meal?

Not at all pleasant *Very pleasant*

|---|

7. How PLEASANT was the AROMA of this meal?

Not at all pleasant *Very pleasant*

|---|

Thank you very much for your cooperation.

Satiety survey

INSTRUCTIONS FOR PARTICIPANTS:

Please read each question carefully and place a mark on the line at the point that best represents your **current feeling**.

Appetite and Sensory Perceptions (Non-Quantified VAS Format)

Please place a mark (X) anywhere along the line that best reflects how you feel **right now**.

---

1. How HUNGRY do you feel right now?

Not at all hungry *Very hungry*

|---|

---

2. How FULL do you feel right now?

Not at all full *Very full*

|--|

3. How STRONG is your desire to eat right now?

No desire at all *Very strong desire*

|--|

4. How much FOOD could you eat right now until you feel satisfied?

Nothing at all *A large amount*

|--|

5. How THIRSTY do you feel right now?

Not at all thirsty *Very thirsty*

|--|

6. How much NAUSEA do you feel right now?

No nausea at all *Severe nausea*

|--|

7. How BLOATED do you feel right now?

Not at all bloated *Very bloated*

|--|

8. How HEAVY do you feel right now?

Not at all heavy *Extremely heavy*

|--|

9. How much HEARTBURN do you feel right now?

No heartburn at all *Severe heartburn*

|--|

10. How strong is your NEED TO DEFECATE right now?

No need at all *Urgent need*

|--|

11. How strong is your APPETITE FOR A MEAL right now?

No appetite at all *Strong appetite*

|--|

12. How strong is your CRAVING FOR SOMETHING SALTY right now?

No craving at all *Strong craving*

|--|

13. How strong is your CRAVING FOR SOMETHING SWEET right now?

No craving at all *Strong craving*

|--|

Thank you very much for your cooperation.
